# *Trans*-cyclooctene—a Swiss army knife for bioorthogonal chemistry: exploring the synthesis, reactivity, and applications in biomedical breakthroughs

**DOI:** 10.1186/s41181-024-00275-x

**Published:** 2024-06-06

**Authors:** Karuna Adhikari, Maarten Vanermen, Gustavo Da Silva, Tim Van den Wyngaert, Koen Augustyns, Filipe Elvas

**Affiliations:** 1 Laboratory of Medicinal Chemistry, University of Antwerp, Antwerp, Belgium; 2 Molecular Imaging and Radiology, University of Antwerp, Antwerp, Belgium; 3 Department of Nuclear Medicine, Antwerp University Hospital, Edegem, Belgium

**Keywords:** *Trans*-cyclooctene, Bioorthogonal chemistry, Pretargeted imaging and therapy, In vivo ligation

## Abstract

**Background:**

*Trans*-cyclooctenes (TCOs) are highly strained alkenes with remarkable reactivity towards tetrazines (Tzs) in inverse electron-demand Diels–Alder reactions. Since their discovery as bioorthogonal reaction partners, novel TCO derivatives have been developed to improve their reactivity, stability, and hydrophilicity, thus expanding their utility in diverse applications.

**Main body:**

TCOs have garnered significant interest for their applications in biomedical settings. In chemical biology, TCOs serve as tools for bioconjugation, enabling the precise labeling and manipulation of biomolecules. Moreover, their role in nuclear medicine is substantial, with TCOs employed in the radiolabeling of peptides and other biomolecules. This has led to their utilization in pretargeted nuclear imaging and therapy, where they function as both bioorthogonal tags and radiotracers, facilitating targeted disease diagnosis and treatment. Beyond these applications, TCOs have been used in targeted cancer therapy through a "click-to-release" approach, in which they act as key components to selectively deliver therapeutic agents to cancer cells, thereby enhancing treatment efficacy while minimizing off-target effects. However, the search for a suitable TCO scaffold with an appropriate balance between stability and reactivity remains a challenge.

**Conclusions:**

This review paper provides a comprehensive overview of the current state of knowledge regarding the synthesis of TCOs, and its challenges, and their development throughout the years. We describe their wide ranging applications as radiolabeled prosthetic groups for radiolabeling, as bioorthogonal tags for pretargeted imaging and therapy, and targeted drug delivery, with the aim of showcasing the versatility and potential of TCOs as valuable tools in advancing biomedical research and applications.

## Background

*Trans-*cyclooctenes (TCOs) are a class of planar chiral alkenes that exhibit unusual reactivity (Ziegler and Wilms 1950; Cope et al. 2002 , 2002), Their structural features have been extensively studied in physical organic chemistry. TCO exhibits high stability towards racemization with an energy barrier of 35.6 kcal/mol (Cope and Pawson 2002). The most stable crown conformation of TCO resembles a stable chair conformation of cyclohexane with an alternating arrangement of equatorial and axial hydrogens (Bach et al. 1972; Allinger et al. 2002; Pigga and Fox 2020). The severely distorted double bond in TCO results in a relatively high energy state of the highest occupied molecular orbital (HOMO). (Cope and Pawson 2002; Barrows and Eberlein 2005; Ermer 1974). This distinctive electronic structure imparts unusual reactivity to TCOs in cycloaddition reactions controlled by the HOMO of an alkene (Weyler et al. 1972; Palacios et al. 1993, 1995; Shea and Kim 1992). Due to their high reactivity profile, TCOs undergo fast ligation reaction with tetrazines. Hence, TCOs are widely employed as bioorthogonal labeling reagents in the domain of bioorthogonal chemistry through reaction with tetrazines for numerous applications (Handula et al. 2021; Pagel 2019; Haiber et al. 2021; Oliveira et al. 2017).

The concept of “bioorthogonal chemistry” refers to a group of chemical reactions which takes place within living organisms under physiological conditions without interacting or interfering with the host organism’s biological system (Sletten and Bertozzi 2011, 2009; Agard et al. 2004; Saxon and Bertozzi 2000). These reactions allow for the modification of biomolecules with non-native functional groups through covalent bond formation and are often used to study biological processes. According to the work done by the Bertozzi Group, a perfect bioorthogonal reaction should fulfill the following four requirements: (i) the reaction should take place under physiological conditions, (ii) the reaction should be selective and inert towards the biological system, (iii) the reaction should have fast kinetics at low concentrations, and (iv) the reaction should not contain any natural functional groups found in biological systems (Sletten and Bertozzi 2011). Although several bioorthogonal reactions have been described, not all of them simultaneously fulfill each of these conditions. Nevertheless, click reactions provide fast reaction rates at mild conditions and low concentration, facilitating PET tracer synthesis for in vivo applications. Among the bioorthogonal reaction, copper-catalyzed azide-alkyne cycloaddition (CuAAC), strain-promoted [3 + 2] azide-alkyne cycloaddition (SPAAC) and inverse electron-demand Diels–Alder (IEDDA) cycloaddition reactions have overwhelmingly dominated the field of radiochemistry. Alternative methods based on photo-click reactions such as photo-click cycloaddition between 9,10-phenanthrenequinones and substituted alkenes have also emerged for radiosynthesis. (Fu et al. 2021) Although promising, photo-click reactions often lack functional group tolerance or require integration of flow photo-microreactor in automated modules, which can be challenging. An overview of different click-reactions used for radiochemistry applications has recently been reviewed by Bauer et al. (2023)

The inverse electron-demand Diels–Alder (IEDDA) cycloaddition reaction between TCO and tetrazine (Tz) is considered by many the optimal bioorthogonal reaction for in vivo applications. This preference stems from its remarkably fast reaction kinetics, with very high reported rate constants (10^7^ M^−1^s^−1^) in physiological conditions (Ravasco and Coelho 2020). Subsequently, the use of TCO-Tz ligation has become prevalent in several fields such as chemical biology, biomedical imaging, radiochemistry, and material sciences (Handula et al. 2021; Pagel 2019; Haiber et al. 2021; Oliveira et al. 2017; Altai et al. 2017). Particularly, the fast reaction kinetics of TCO with tetrazines has garnered significant attention in nuclear medicine where the TCO moiety has been actively explored as a prosthetic group for introduction of a radioisotope to a vector molecule or as a bioorthogonal tag for subsequent labeling of a target of interest for pretargeted imaging and therapy. A promising application of the pretargeting approach is cancer diagnosis and therapy, allowing the use of long circulating vectors at optimal tumor-to-background ratios, thereby minimizing the radiation damage to non-targeted tissues.

This review presents a comprehensive overview of the recent progress in the field of TCO development, specifically focusing on its role as a crucial reactant in the IEDDA reaction. We revisit the mechanisms that govern their reactivity and present the synthesis of TCOs and their derivatives. Furthermore, we emphasize the wide range of applications of TCOs in the fields of biomedical imaging, radioimmunotherapy, and targeted drug delivery. By exploring these aspects, this review aims to provide valuable insights into the current state-of-the-art in TCO research and its implications in various scientific disciplines.

## Main text

### Inverse *electron*-demand Diels–Alder (IEDDA) reaction and mechanism

**Scheme 1:**
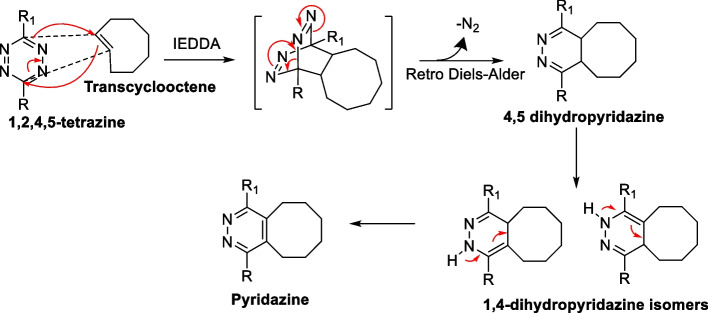
Reaction mechanism of inverse electron-demand Diels–Alder [4 + 2] (IEDDA) between a trans-cyclooctene and 1,2,4,5-tetrazine. The cycloaddition is followed by a retro Diels–Alder with expulsion of N_2_ forming 1,4-dihydropyridazine isomers which further oxidizes to pyridazine

The reactivity of Tzs with unsaturated compounds has been previously elucidated by Carboni and Lindsey (1959) However, the initial discovery of IEDDA as a bioorthogonal ligation was reported by Blackman et al. (2008) and Devaraj et al. (2008) The IEDDA reaction proceeds through a [4 + 2] cycloaddition between an electron-rich dienophile (alkene or alkyne) and electron-poor diene (1,2,4,5-tetrazine) forming a highly strained bicyclic intermediate. Subsequently, a retro-Diels–Alder reaction occurs with N_2_ evolution, resulting in the formation of a stable six-membered 4,5-dihydropyridazine ring. When reacted with an alkene dienophile, 4,5-dihydropyridazine isomerizes to 1,4-dihydropyridazine isomers or oxidizes to form pyridazine. In contrast, alkyne dienophiles undergo direct transformation, leading to the formation of pyridazines (Scheme 1).

**Fig. 1:**
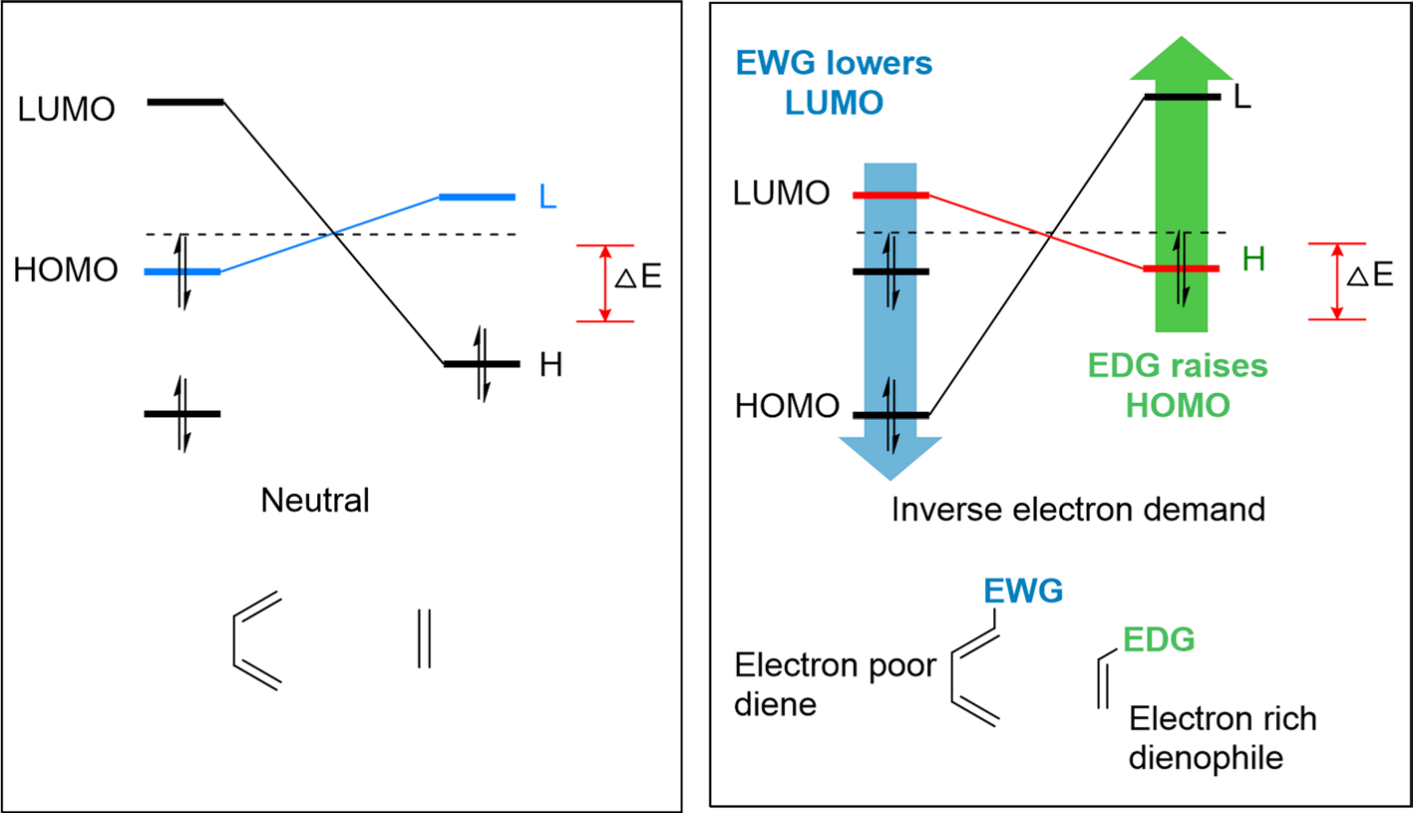
Frontier molecular orbital (FMO) model of cycloaddition for a Diels–Alder cycloaddition reaction and IEDDA with ∆E depicting the energy gap between the orbitals. In IEDDA, the energy gap between the highest occupied molecular orbital (HOMO) of the dienophile and the lowest unoccupied molecular orbital (LUMO) of the diene is influenced by the presence of electron-donating groups (EWG) on the diene and electron-withdrawing groups on the dienophile. Adapted from Oliveira et al. (2017) with permission from Royal Society of Chemistry

Several factors influence the rate of the IEDDA reaction. A faster reaction rate can be achieved by decreasing the energy gap between the dienophile’s HOMO and the diene’s lowest unoccupied molecular orbital (LUMO; Fig. 1) (Oliveira et al. 2017). Additionally, steric effects, intramolecular repulsions, lower distortion energies of the reaction partners, a strain effect on the dienophile, and the position and nature of the substituents on the reaction partner can also impact the reaction kinetics. The subsequent section explores the various parameters that can affect the kinetics of IEDDA.

#### Reactivity of the tetrazines

**Fig. 2:**
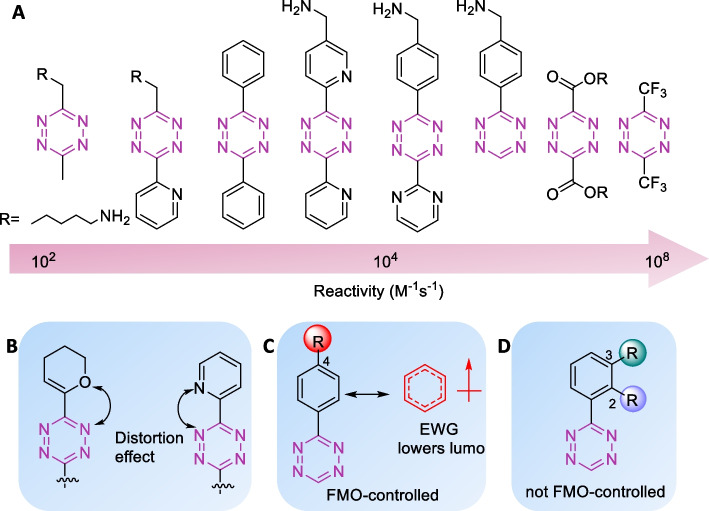
Structure and reactivity of tetrazine influenced by electronic, steric, and distortion effects. (**a**) Tetrazines in increasing order of reactivity. The EWG groups increase the reactivity of the tetrazines. **b** Repulsive intramolecular interactions reduce the Tz distortion energy and increase reactivity. **c** The reactivity of 4-substituted tetrazines is FMO-controlled and the reactivity increases in presence of EWG **d** reactivity of 2-substituted or 3-substituted tetrazines is not FMO-controlled

Tzs are the most common dienes used in IEDDA reactions. The substitutions performed on the tetrazine backbone can influence its reactivity. Electron-withdrawing groups (EWG) decrease the energy level of the LUMO of the diene, thereby decreasing the HOMO_*Dienophile*_–LUMO_*Diene*_ energy gap. Thus, the reactivity of tetrazines can be increased by installing EWG on the tetrazine scaffold (Fig. 1). Computational and experimental studies on the kinetics of 3,6-disubstituted-Tzs showed that the tetrazines with the lowest LUMO energy levels were the most reactive (Boger et al. 1998). Consequently, Tzs-containing EWGs such as carboxylates, pyridines, and pyrimidines react faster than tetrazines substituted with electron-donating methoxy or methyl groups (Karver et al. 2011; Kele et al. 2015). However, Tz-bearing strong EWGs are unstable in aqueous conditions, which can be a limiting factor for in vivo ligation reactions. From a structural point of view, diaryl Tzs exhibit a favorable balance between reactivity and stability. Steric effects also influence the reactivity of Tzs as hydrogen-substituted Tz show a 70-fold increased reactivity than di-substituted Tzs (Karver et al. 2011; Kele et al. 2015). Given the enhanced reactivity of H-Tzs together with their favorable stability, H-Tzs are often used for in vivo and in vitro applications (Devaraj et al. 2008, 2009; Agustin et al. 2016; Arsic et al. 2022; Battisti et al. 2021; Beliu et al. 2019; Cook et al. 2016, 2022; Garcia-Vazquez et al. 2021). Furthermore, unfavorable steric repulsions were reported when Tzs were substituted with bulky *tert*-butyl groups (Yang et al. 2014). A study by Svatunek et al. (2022) showed that repulsive intramolecular interactions on substituted Tz lower their distortion energy which increases their reactivity towards dienophiles. Further studies by Battisti et al*. *(2022) revealed that the reactivity is not only determined by the type of substituents but also by their position on the aromatic ring attached to Tzs (Fig. 2). Although the pH of the solution has a minor effect on the reaction rate, the interaction with solvents should also be considered. Protic solvents can stabilize the interaction of the activated complex while enhancing the hydrophobic interactions between the cycloaddends, which accelerates the reaction rate. Also, the hydrogen bonding of water with Tzs accelerates the reaction rate (Battisti et al. 2022; Wijnen et al. 1996; Meijer et al. 1998).

#### Reactivity of dienophiles

In contrast to a Diels–Alder cycloaddition, in a IEDDA reaction dienophiles exhibit increased reactivity when substituted with electron-donating groups (EDG). The energy levels of both the HOMO and LUMO orbitals of the dienophile are increased by EDG, which results in a decreased HOMO_dienophile_-LUMO_diene_ energy gap (Fig. 1) (Oliveira et al. 2017). The effect of electronic substituents on unstrained dienophiles has been extensively studied, where electron-rich groups favor faster kinetics. Sauer and coworkers investigated the reaction kinetics of various Tzs and dienophiles and established that the greater the ring strain (cyclopropene > cyclobutene > cyclopentene > cyclohexene > cyclooctene), the faster the dienophiles react with dienes (Thalhammer et al. 1990). However, the strain effect is the most crucial factor affecting dienophile reactivity. Computational analysis by Liu et al. (2013a) revealed that strained dienophiles display a pre-distorted conformation similar to their transition state structures, requiring less distortion energies for the reaction to occur with dienes. Among dienophiles, TCO is the most reactive and displays 7 times higher reactivity than *cis*-cyclooctene (CCO) (Thalhammer et al. 1990). TCO shows a remarkable increase in reactivity in comparison to cyclic alkenes with higher ring strain such as cyclopropene (Bach 2009). Computational studies have shown that TCO adopts a energetically more favorable ‘crown’ conformation giving it a higher reactivity profile than the ‘half-chair’ conformation of *cis*-cyclooctene (Barrows and Eberlein 2005; Bach 2009; Leong et al. 1998; Liu et al. 2014).

**Fig. 3:**
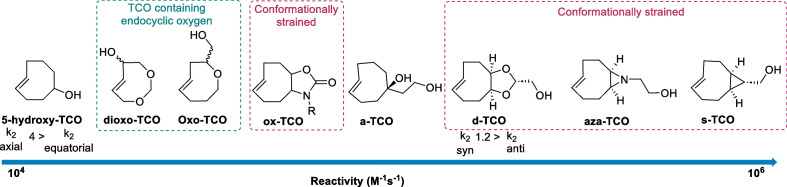
Comparison of the reactivities of different TCO derivatives used in ligation reactions. The reactivity is enhanced by increasing the ring strain via cis-ring fusion to the cyclooctene core. Stereochemistry and the inclusion of endocyclic or exocyclic heteroatoms also influence the reactivity

In recent years, computational studies have provided additional insight into the design of reactive dienophiles. Taylor et al. demonstrated that the reactivity of dienophiles can be increased by introducing conformational strain through *cis*-ring fusion on the eight-membered ring and designed a TCO with a *cis*-fused cyclopropane ring (s-TCO), which enforced a highly strained half-chair conformation. Transition-state calculations indicated that s-TCO in the half-chair conformation would react faster to the crown conformation of TCO, with a predicted ∆∆G ‡ value of 3.34 kcal/mol. When experimentally determined, the s-TCO showed ∆∆G ‡ value of 3.0 kcal/mol and reacted 160 times faster with 3,6-diphenyl-s-tetrazine than the original TCO. (Taylor et al. 2011) Similarly, *cis*-dioxolane fused *trans*-cyclooctene (d-TCO) was designed to increase the stability and hydrophilicity while retaining the highly strained ‘half-chair’ conformation, which showed 27-fold rate enhancement in comparison to the original TCO (Fig. 3) (Darko et al. 2014).

The reactivity of strained dienophiles is also determined by the stereochemistry (Wagner et al. 2015). As demonstrated by density functional theory (DFT) calculations, the axial isomer of functionalized TCO was found to be 1.1 kcal/mol higher in energy than the corresponding equatorial isomer (Wagner et al. 2015). Experimentally, the reactivity of the axial isomer was found to be four times greater than that of the equatorial isomer (Darko et al. 2014; Hoffmann et al. 2015; Rossin et al. 2013a). Similarly, the *syn*-diastereomer of d-TCO showed a higher rate constant compared to the *anti*-diastereomer (~ 1.2 x) (Darko et al. 2014). Similar trends were also observed for other dienophiles, such as norbornene and bicyclononynne (BCN), which has been discussed in detail elsewhere (Oliveira et al. 2017).

### Synthesis of *trans*-cyclooctenes

**Scheme 2:**
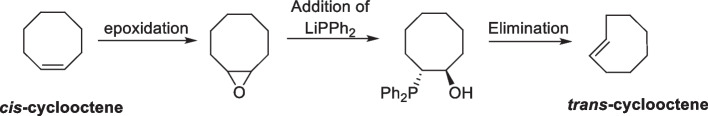
Multistep synthesis of trans-cyclooctene from cis-cyclooctene via inversion of the alkene stereochemistry

TCO was first synthesized in 1950 through the Hoffman elimination of trimethylcyclooctyl ammonium iodide, resulting in a mixture with *cis*-cyclooctene (Ziegler and Wilms 1950). In 1953, separation of TCO was achieved by forming a water-soluble TCO ⋅AgNO_3_ complex, which was then decomplexed with NH_4_OH to yield the pure compound (Cope et al. 2002). Although there are well-known methods for synthesis of TCO, the synthetic route for functionalized derivatives was less described (Reese and Shaw 1970; Braddock et al. 2004; Whitham et al. 1971 , 1971). Synthesis of TCO from CCO via stereospecific methods has also been described (Hines et al. 1973; Corey and Shulman 1968; Vedejs et al. 1973; Vedejs and Fuchs 2002). An illustrative example involves a sequence of epoxidation steps of the double bond, subsequent addition of LiPPh_2_, and ultimately elimination, resulting in the pure inverted *trans*-alkene as a product (Scheme 2) (Bridges and Whitham 1974). However, such protocol requires a complex multistep synthesis procedure and harsh reaction conditions, limiting the scope of TCO derivatives that can be prepared.

Royzen et al. (2008) described a one-step closed-loop flow photoisomerization procedure for converting CCO to TCO. Currently, this procedure stands as the most commonly used method for obtaining TCO derivatives. Novel synthetic routes have been established for the synthesis of TCO. A study by Wang et al. (2014) and Wang et al. (2015a) reported the preparation of TCOs by 4-π electrocyclic ring opening (Ito et al. 2019). Similarly, the synthesis of heteroatom-containing *trans*-cycloalkenes have been reported (Lambert et al. 2017). Prevost et al. (2010) described the synthesis of *trans*-dioxasilacyclooctenes, while Tomooka et al*. *(2014) introduced the dialkoxysilane cyclooctene (Machida et al. 2018). Recently, Pigga et al. (2021) reported a synthetic route to axial TCOs using a stereo-controlled additions of nucleophiles to *trans*-cyclooct-4-enone. Similarly, more accessible synthetic pathways towards click-cleavable TCO-linker have also been reported (Wilkovitsch et al. 2020; Kuba et al. 2023; Liu et al. 2023; Sondag et al. 2023).

### Photochemical isomerization of *cis*-cyclooctene to *trans*-cyclooctene

**Scheme 3:**
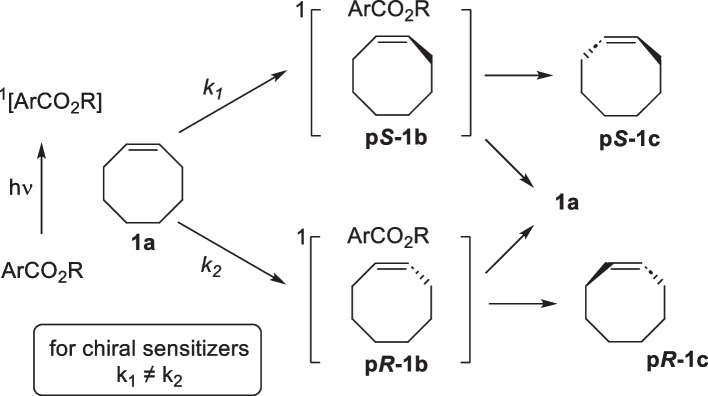
Scheme depicting the formation of chiral exciplexes when cis-cyclooctene is irradiated in the presence of a chiral sensitizer. Reproduced from Pigga and Fox (2020) Copyright 2019 Wiley–VCH Verlag GmbH & Co. KGaA, Weinheim

The photoisomerization of *cis*-cyclooctene in the presence of a singlet sensitizer represents a direct route for the conversion of CCO to TCO (Maeda et al. 2011). Maeda et al. (2011), Kaneda et al. (2002), Inoue et al. (1999), Shi and Inoue (1998), Inoue et al. (1998), Tsuneishi et al. (1993), Inoue et al. (2002); Inoue et al. (1978) and Inoue et al. (1983) first described the enantioselective photoisomerization of several *cis*-cycloalkenes into their *trans*-isomers, where chiral aromatic esters were found to be the most optimal sensitizers. Chiral aromatic esters form a singlet excited state when irradiated at 254 nm. The singlet excited state of the sensitizer interacts with CCO to form diastereomeric exciplexes (pS-1b and pR-1b; Scheme 3). Thereafter, the “twisted singlet” exciplexes disassociate into the respective TCO and CCO (Scheme 3) The photoisomerization process allows for an enantioselective synthesis due to a difference in the formation rates of the two "twisted singlet" exciplexes with chiral sensitizers.

The photochemical synthesis of functionalized *trans*-cyclooctenes showed, unfortunately, limitations in terms of low yields, with a maximum of 23% conversion. When using a functionalized TCO, the formation of diastereomers was observed, which further decreased the yield of the desired product (Blanco-Ania et al. 2018). Moreover, photodegradation of functionalized TCO was also observed as the yield dropped to < 5% when irradiated up to 18h (Royzen et al. 2008).

**Fig. 4:**
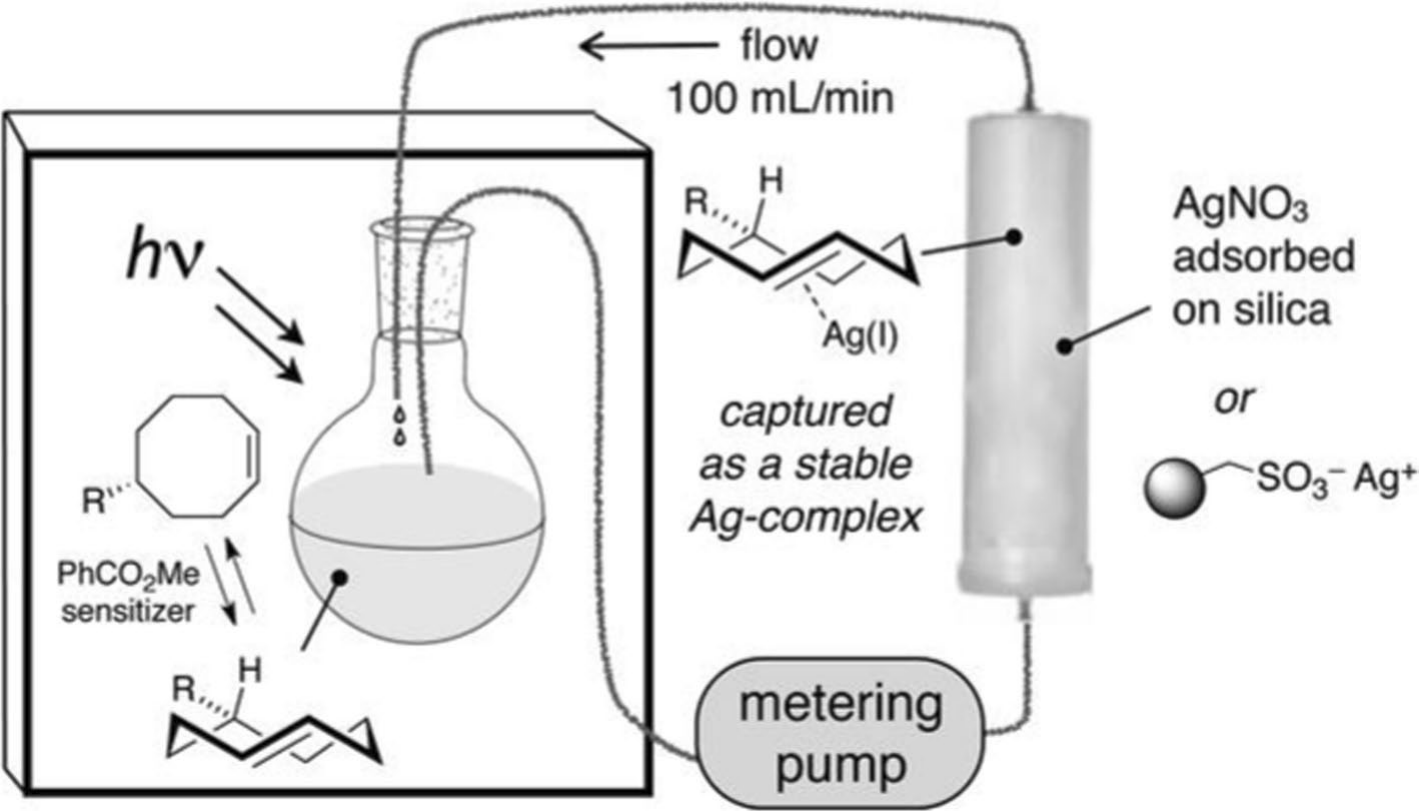
Schematic of Flow-Photo isomerization apparatus for the synthesis of trans-cyclooctene. Adapted with permission from Darko et al. (2018) Copyright 2018 Georg Thieme Verlag KG

The flow-photoisomerization strategy introduced by Royzen et al. (2008) is based on the selective metal complexation of the *trans*-isomer. TCO interacts strongly with metals owing to the strain relief in the hydrocarbon framework, which requires minimal energy for reorganization (Cedeño and Sniatynsky 2005; Mander and Williams 2016). As originally reported, the setup consists of a closed-loop photoreactor, where a solution of CCO and methyl benzoate are irradiated in a quartz flask at 254 nm. The reaction mixture is pumped continuously through a cartridge packed with AgNO_3_-impregnated silica gel during irradiation. The TCO derivative selectively binds to AgNO_3_-impregnated silica, while the *cis* isomer is recirculated back to the reaction flask for photoisomerization (Fig. 4). Once the CCO is completely consumed, the silica is removed and treated with NH_4_OH or NaCl for base-sensitive compounds to release TCO from AgNO_3_ (Royzen et al. 2008). The TCO derivative is subsequently recovered by extraction. This innovative strategy provides a selective and efficient means of obtaining TCO derivatives, opening new possibilities for their application in various chemical processes.

Since its original description, flow photoisomerization has been used by several research groups for the synthesis of TCO for different applications (Hoffmann et al. 2015; Sanzone and Woerpel 2016; Siegl et al. 2019, 2018; Kara et al. 2019; Dadhwal et al. 2019; Versteegen et al. 2018; Gracht et al. 2018; DeMeester et al. 2018; Bruins et al. 2018; Bernard et al. 2018; Vazquez et al. 2017; Marjanovic et al. 2017; Maggi et al. 2016; Lorenzo et al. 2016; Kozma et al. 2016; Denk et al. 2016; Mohamed et al. 2016; Wang et al. 2015b; Blizzard et al. 2015; Wollack et al. 2014; Mejia Oneto et al. 2014). While the flow procedure has proven effective, some modifications and alternative setups have been explored. One approach involves mimicking flow photoisomerization by periodically stopping irradiation, filtering the reaction mixture through AgNO_3_-impregnated silica to capture the TCO, and reusing the filtrate for photoisomerization (Devaraj et al. 2009; Schoch et al. 2012). Although this avoids the cost of flow equipment, it is more labor intensive, and the yields are lower. Another variation of the setup used a flow cell consisting of a quartz tube placed between two UV light sources in combination with a reservoir flask containing the reaction solution. The reaction solution was pumped continuously through a UV-irradiated quartz tube. This approach enabled upscaling of the reaction at low cost, as reservoir flasks of any size and shape could be used without the need for different quartz flasks (Svatunek et al. 2016). Billaud et al. reported a microfluidic device in which two microreactors wrapped around a UV lamp were used in parallel with several beds of AgNO_3_-impregnated silica, which could be exchanged during irradiation to avoid saturation. With this procedure, high conversion of CCO to TCO was achieved in a short time (Billaud et al. 2017). Fox et al. also modified their setup using Ag(I) immobilized on Tosic-acid-functionalized silica gel (TAg silica). TAg silica allows for higher loading of silver without the risk of silver leaching enabling higher concentration of TCO trapping as the metal covalently bound to the solid support. This facilitated the scaling up of photoisomerization of TCO derivatives with substitutions such as hydroxyl, carboxylic acid, hydroxyethylether, diol, and protected amines (Darko et al. 2018).

A low-cost and readily accessible apparatus for photoisomerization has recently been described. This setup incorporates the use of fluorinated ethylene propylene (FEP) tubing coiled on a household germicidal lamp inside an aluminum vent pipe. The reaction solution was connected to a flash chromatography system and pumped continuously through the FEP tubings (Pickel et al. 2021). This make-shift apparatus yielded 48–76% of different TCOs and avoided the high cost of the photoreactors.

A recent technique developed by Blanco-Ania et al. (2018) applied a liquid–liquid extraction method. This method involves a continuous flow of UV-irradiated CCO in heptane over an aqueous AgNO_3_ solution. The organic phase containing CCO flowed through an FEP tubing coiled around the UV lamp (254 nm) and into the AgNO_3_ aqueous solution. In this process, TCO is trapped in the aqueous solution, whereas CCO returns to the organic phase. This enables the upscaling of the photoisomerization reaction as it allows for the external addition of CCO, maintaining a consistent concentration of CCO. The authors reported up to 2.2 g/h of TCO produced and the setup could be applied for the synthesis of several commonly used TCOs (Blanco-Ania et al. 2018). This efficient and scalable technique represents a valuable advancement in the field of TCO synthesis, facilitating large-scale production of TCO derivatives.

### *Trans*-cyclooctenes in ligation reactions

The following section summarizes the various TCO derivatives that have been used for IEDDA ligation reactions over the years.

#### *Trans*-cyclooct-4-enol and its derivatives

**Scheme 4:**
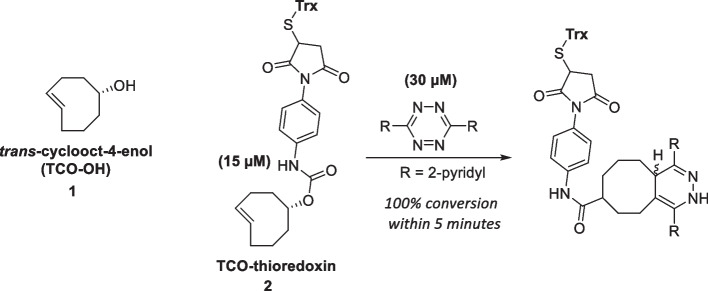
Structure of trans-cyclooct-4-enol and the reaction between thioredoxin-derivatized TCO-OH and 3,6-dipyridyl-s tetrazine. Adapted with permission from Blackman et al. (2008) Copyright 2008, American Chemical Society

Blackman et al. reported the very fast kinetics of parent TCO with electron-deficient Tzs (k_2_ = 2.0 × 10^3^ M^−1^s^−1^. This study demonstrated the bioorthogonality of the reaction as it proceeded successfully in organic solvents, water, cell media, or cell lysates in quantitative yield (Blackman et al. 2008). The group synthesized an alcohol-functionalized derivative ***trans*-cyclooct-4-enol** (TCO-OH; 1) through the photoisomerization of *cis*-cyclooct-4-enol, which is the most commonly used TCO derivative for biological applications. In the initial investigation by Blackman et al. (2008), ***trans*-cyclooct-4-enol** was further derivatized with thioredoxin (Trx) and reacted with 2 × excess Tz, and complete consumption of the reagents was observed within 5 min, illustrating the biocompatibility of TCO-Tz ligation for the conjugation of proteins (Scheme 4).

Based on these results, Rossin et al. studied the potential use of TCO-tetrazine ligation under more demanding in vivo conditions, where metabolism, side reactions, and prolonged residence times play an important role. TCO conjugated to CC49 antibody (mAb) showed a second-order rate constant of (13 ± 0.08) × 10^3^ M^−1^s^−1^ with [^111^In]In-labeled-Tz in PBS at 37 °C. Importantly, 75% of the TCO bound to CC49 remained reactive in vivo after 24 h. However, slow deactivation (25% in 24 h) of TCO in serum was observed. This, in turn, limited the lag time between the mAb conjugate and Tz probe administration for the in vivo pretargeted imaging study (Rossin et al. 2010). In a follow-up study, the sole mechanism of deactivation of mAb-bound TCO in serum was found to be the *trans-*to*-cis* isomerization mediated by copper-containing serum proteins (Rossin et al. 2013a). This was circumvented by shortening the linker distance between the TCO and the lysine residue on mAb, which prevented interaction with serum proteins by increasing the steric hindrance on the TCO. Additionally, they also demonstrated that the axial substituent is up to fourfold more reactive due to the increased strain in axially substituted isomers (Rossin et al. 2013a). When reacted with 3,6-dipyridyl-s-tetrazine in aqueous media, the measured k_2_ values for the equatorial and the axial diastereomer were (22 ± 0.04) × 10^3^ M^−1^s^−1^ and (80 ± 0.2) ×  10^3^ M^−1^s^−1^, respectively (Darko et al. 2014).

Other ring-substituted isomers have also been reported previously. TCO—based noncanonical amino acids (ncAAs) have been genetically encoded using the double-mutant synthetase MmPylRS (Y306A, Y384F) for site-specific labeling of proteins in a short time for super-resolution microscopy (Plass et al. 2012). However, the genetic encoding of lysine-modified *trans*-cyclooct-4-enol is unsatisfactory. Lemke and coworkers explored the ***trans*-cyclooct-2-enol** and ***trans*-cycloocte-3-enol** ring isomers as an alternative (Nikic et al. 2014). The lysine-modified 3-enol and the 2-enol amino acid isomers showed significantly higher genetic incorporation into green fluorescent protein (GFP) in *Escherichia coli* expression cultures by the mutant tRNA synthetase than the 4-enol isoform. Their studies revealed that a diastereomeric mixture of the 3-enol isomer displayed reaction kinetics similar to that of the 4-enol isomer. However, the 2-enol isomer showed slower kinetics but a tenfold increase in stability, showing a lower level of *trans to cis* isomerization (Nikic et al. 2014). In a follow-up study, the authors reported a higher activity of the axial isomer of *trans*-cyclooct-2-enol when compared to the equatorial isomer, just as *trans*-cyclooct-4-enol (Hoffmann et al. 2015). Notably, the bicyclic product of the [4 + 2] cycloaddition reaction of *trans*-cyclooct-2-enol with Tz undergoes β-elimination, releasing the conjugated lysine bond under certain conditions. This property was further exploited as click-to-release strategy for targeted drug release which will be referred to later in this review (Versteegen et al. 2013).

#### Axial-5-hydroxy-*trans*-cyclooctene (a-TCO)

**Scheme 5:**
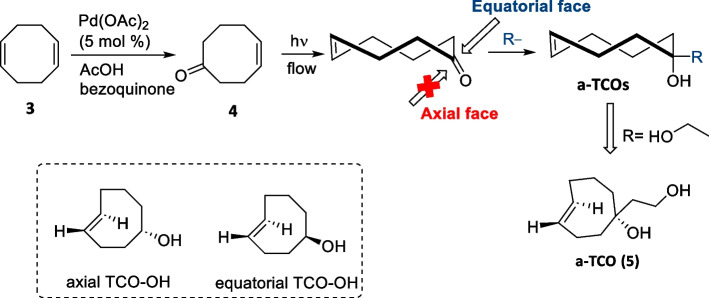
Diastereoselective synthesis of a-TCO via nucleophilic addition is preferred at the equatorial face. Structures of axial and equatorial TCO-OH. Adapted with permission from Pigga et al. (2021) Copyright 2021 Wiley–VCH GmbH

The widely used photoisomerization process generates equatorial and axial diastereomers in a 2.2:1 ratio, yielding the axial isomer in low isolated yield (≤ 24%), necessitating a tedious separation process. For some TCO derivatives, the separation of both diastereomers by flash chromatography can be challenging or not feasible. Pigga et al. devised a diastereoselective synthetic route to TCOs via the addition of nucleophiles to *trans*-cyclooct-4-enone in a stereocontrolled manner. This approach addressed the problem of low stereoselectivity during TCO synthesis. The preparation of *trans*-cyclooct-4-enone (4) proceeded in two steps via the Wacker oxidation of 1,5-cyclooctadiene, followed by photoisomerization. As ketone (4) lacks a stereocenter, it can be isomerized without complications related to diastereoselectivity. Transition state calculations predicted that nucleophilic addition to ketones occurs exclusively from the equatorial face, resulting in the formation of only axial-substituted diastereomers. Experimentally, the nucleophilic addition of hydride exclusively afforded the axial diastereomer (Scheme 5) (Pigga et al. 2021). The described route shows significant advantages for the synthesis of TCOs as it is short, selective, and scalable. Several a-TCO conjugates were prepared from the central ketone intermediate. Diol-derivatized a-TCO (5) showed rapid kinetics with a 3,6-dipyridyl-s-tetrazine derivative with a rate constant (k_2_) of (150 ± 8) ×  10^3^ M^−1^s^−1^, which is two times faster than that of the axial diastereomer of ***trans*-cyclooct-4-enol** (k_2_ = (70 ± 2) ×  10^3^ M^−1^s^−1^. The steric effect due to geminal substitution on the TCO core led to increased olefinic strain in a-TCO and resulted in faster kinetics. The a-TCO (5) was also predicted to show improved hydrophilicity when compared to other TCO derivatives with a lower calculated logP (cLogP) value of 1.11 when compared to TCO (cLogP 1.95), oxo-TCO (cLogP 1.33) and d-TCO (cLogP 1.76). When applied in a cell-imaging assay, the increased hydrophilicity of a-TCO allowed for a rapid washout of the fluorophore-conjugated a-TCO leading to lower background fluorescence when compared to the less hydrophilic oxo-TCO (Pigga et al. 2021).

#### Conformationally strained *trans*-cyclooctene (s-TCO) and derivatives

**Scheme 6:**
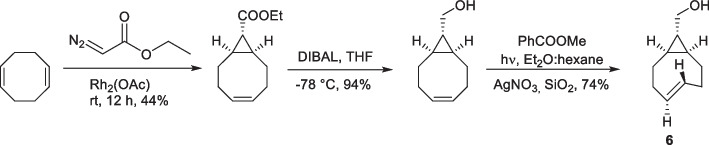
Synthesis of conformationally strained s-TCO

Computational studies have increased our understanding of the rapid kinetics of TCO and have provided insights into their development. Ab initio calculations by Bach (2009) found the strain energy of half chair conformation of a TCO to be 5.9 kcal/mol greater in energy than the crown conformation (0 kcal/mol). Based on their increased strain energy, non-crown conformers were speculated to accelerate the reactivity towards Tzs. Dommerholt et al. (2010) used a cyclopropane-fused strained cycloalkyne (BCN) which showed higher reactivity towards azide in the SPAAC reaction. In parallel, Fox and coworkers demonstrated that introducing a conformational strain on TCO with a fused cyclopropane ring causes the alkene in the bicyclic system to adopt a highly strained half-chair conformation, increasing the TCO reactivity. The strained TCO annealed to a cyclopropane with *cis-*ring fusion was termed s-TCO (Taylor et al. 2011). The group synthesized an alcohol-functionalized analog of s-TCO (6) via cyclopropanation of 1,5-cyclooctadiene with ethyl diazoacetate, followed by ester reduction and *cis–trans* photoisomerization (Scheme 6).

**Fig. 5:**
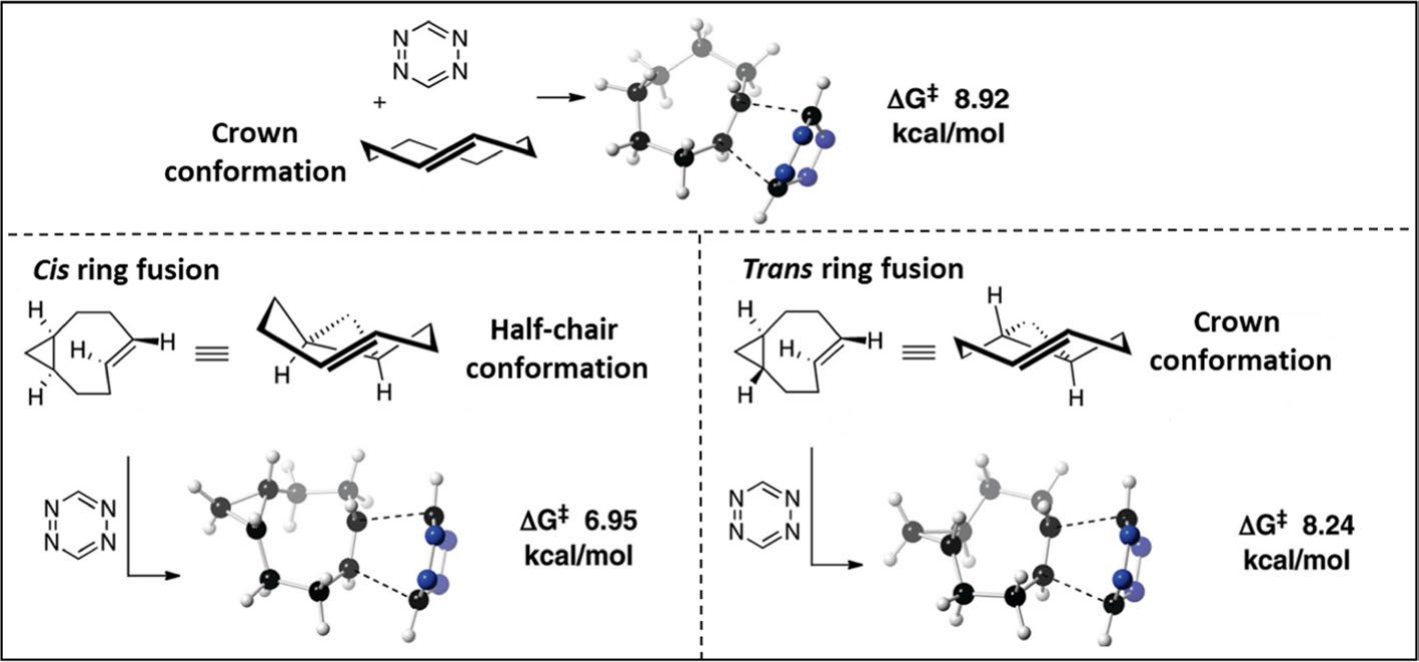
Transition-state structures and respective energy barriers for the Diels–Alder reaction of s-tetrazine with crown conformers of trans-cyclooctene, cis-ring-fused s-TCO, and trans-ring-fused s-TCO. Adapted with permission from Taylor et al. (2011) Copyright 2011, American Chemical Society

To demonstrate that the elevated reactivity of s-TCO is caused by additional ring strain, the reaction barrier between s-tetrazine and *trans*-fused s-TCO was investigated. The calculated reaction barrier for *trans*-ring-fused s-TCO was ΔG^‡^ = 8.24 kcal/mol, which is significantly higher than that of *cis*-ring-fused s-TCO (ΔG^‡^ = 6.95 kcal/mol). A *trans*-ring fusion allows the eight-membered ring to adopt a crown conformation, similar to the parent TCO, thus explaining the difference in reactivity between the two diastereomers (Fig. 5). Specifically, when compared with TCO, the augmented ring strain in s-TCO led to a 160-fold increase in reactivity with 3,6-diphenyl-s-tetrazine (Taylor et al. 2011).

A water-soluble derivative of s-TCO showed a remarkable rate constant (k_2_) of (3,300 ± 40) ×  10^3^ M^−1^s^−1^ with a 3,6-dipyridyl-s tetrazine derivative, making s-TCO the fastest TCO to date (Darko et al. 2014). With its ultra-fast kinetics, s-TCO allows rapid fluorogenic labeling inside living bacteria in combination with tetrazine-modified GFP (Seitchik et al. 2012). However, the inherent reactivity of s-TCO is accompanied by a trade-off in terms of stability. s-TCO isomerizes rapidly in the presence of high thiol concentrations (30 mM) (Taylor et al. 2011; Lang et al. 2012). In vivo, s-TCO conjugated to a mAb showed a half-life of 0.67 days with rapid deactivation into its *cis* isomer (Rossin et al. 2013a). Moreover, these derivatives exhibit limited stability to prolonged storage and must be kept as cold solutions to prevent polymerization and isomerization. Storing these derivatives as stable silver(I) metal complexes can extend their shelf lives (Fang et al. 2019). Additionally, the synthesis lacks stereoselectivity, requiring separation of *syn*- and *anti*-product diastereomers through chromatographic techniques, limiting its widespread use (Darko et al. 2014).

**Scheme 7:**
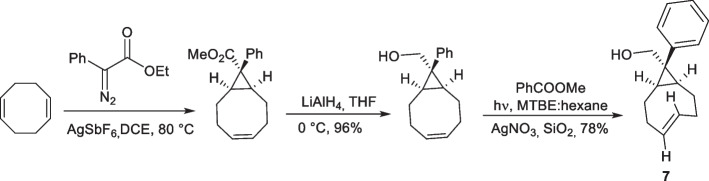
Synthesis of double-functionalized s-TCO (Ravasco et al. 2020)

To incorporate an additional vector for functionalization, Ravasco et al. (2020) modified s-TCO at the cyclopropane moiety (7; Scheme 7). The cyclopropane ring was double-functionalized with an aromatic substituent and an alcohol on a quaternary carbon. DFT calculations of the cycloaddition between Tz and the double-functionalized s-TCO (7) demonstrated that the introduction of an aromatic ring did not have a significant effect on the energy barrier required for the reaction to occur. A ΔG‡ value of 7.0 kcal/mol was observed for the double-functionalized s-TCO (7), which was comparable with that of parent s-TCO (ΔG‡ = 7.1 kcal/mol). Similarly, the distortion energy in the transition state of double-functionalized s-TCO (ΔE_dist_ = 1.6 kcal/mol) remained comparable to parent s-TCO (ΔE_dist_ = 1.7 kcal/mol). In ^1^H NMR competition experiments, double-functionalized sTCO (*k*_rel_ = 1.54 ± 0.1) was estimated to react 1.5 times faster than sTCO (*k*_rel_ = 1) with 3,6-dipyridyl-s tetrazine, confirming the results from the DFT calculations and indicating that the additional linker on the scaffold did not affect the reactivity.

When assessed for stability, double functionalized s-TCO remained stable for over 70 h in deuterated phosphate buffer (pH 7.4 and 6, 1:1 DMSO/PBS) and up to 24 h when exposed to amine nucleophiles (30 mM n-pentylamine in CD_3_OD-*d*_*4*_). Similar to other TCO derivatives, the compound isomerized (12% after 12 h) in the presence of concentrated ethanethiol (30 mM in CD_3_OD-*d*_*4*_) and when exposed to a high concentration of reduced glutathione (GSH) (60% in 12 h in DMSO/PBS 1:1, pH 7.4). Overall, the compound showed a slightly greater stability than s-TCO (Ravasco et al. 2020). In general, the established synthetic pathway presents an opportunity for the incorporation of additional functional groups. However, to enhance the suitability of this compound for biological assays, it may be necessary to incorporate additional polar groups. This is because the s-TCO core comprises a hydrophobic core, which may result in an increased nonspecific uptake in biological environments.

#### *cis*-Dioxolane-fused *trans*-cyclooctenes (d-TCO)

**Scheme 8:**
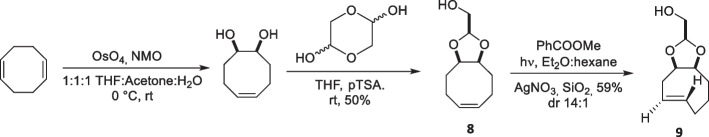
Diastereoselective synthesis of d-TCO

Darko et al. (2014) developed another conformationally strained TCO variant based on computational studies, termed *cis*-dioxolane-fused *trans*-cyclooctene (d-TCO). The incorporation of a *cis*-fused dioxolane ring imposed a strained half-chair conformation of the cyclooctene ring, resulting in enhanced reactivity. The introduction of inductive electron-withdrawing oxygen also increased the hydrophilicity and stability of the compound due to the reduction of electron density from the alkene (Darko et al. 2014). However, this reduction in electron density also lowers the HOMO_dienophile_ resulting in decreased reactivity, creating a compromise between reactivity and stability.

d-TCO derivatives were prepared on a multigram scale through diastereoselective synthesis. Upjohn dihydroxylation of 1,5-cyclooctadiene, followed by dioxolane formation in the presence of an aldehyde, which afforded the *syn* diastereomer (8) in greater yield, which was subsequently photoisomerised to d-TCO (9; Scheme 8). Although slower than s-TCO, d-TCO exhibited excellent reactivity towards tetrazines. Additionally, the functionality of dioxolane enhances the hydrophilicity of the molecule (logP 0.94), allowing kinetic measurements in an aqueous solution. The d-TCO *syn*-diastereomer reacted with a water-soluble derivative of 3,6-dipyridyl-s-tetrazine with a rate constant k_2_ of (366 ± 15) ×  10^3^ M^−1^s^−1^, whereas the *anti*-diastereomer exhibited a k_2_ value of (318 ± 3) ×  10^3^ M^−1^s^−1^ at 25 °C in water. Moreover, the d-TCO derivatives demonstrated improved stability compared to the more reactive s-TCO, showing no decomposition when stored in aqueous solutions at room temperature. No degradation or isomerization of d-TCO was observed in phosphate-buffered D_2_O for up to 14 days. After incubation in human serum at room temperature for four days, the compound remained as a *trans*-isomer (> 97%). Similar to other TCOs, d-TCO is also susceptible to thiol-promoted isomerization (43% after 5h at pH 7.4) (Darko et al. 2014). Due to its improved hydrophilicity, stability, and remarkable kinetics d-TCOs have been a compound of interest in several biological applications.

#### Strained aziridine-fused *trans*-cyclooctene (aza-TCO)

Vazquez et al. (2017) reported the formation of a fluorescent 1,4-dihydropyridazines product following the cycloaddition between an s-Tz and axial *trans*-cyclooct-4-enol. The authors hypothesized the involvement of the axially substituted hydroxy group in the tautomerization of the dihydropyridazine product. Through conformational analysis and DFT modeling, the key step for the formation of fluorescent 1,4-dihydropyridazine was identified as the transfer of a hydrogen atom from one of the bridgehead carbon atoms to a heteroatom on the 4,5-dihydropyridazine intermediate. Importantly, this hydrogen transfer was only feasible when the heteroatom and the hydrogen atom were in close proximity. Notably, this phenomenon was observed for the dihydropyridazine intermediate formed with axially substituted TCO, but not for the equatorial isomer. Additionally, modifications of the alcohol group either altered or resulted in a complete disappearance of fluoroscence (Vazquez et al. 2017).

**Scheme 9:**
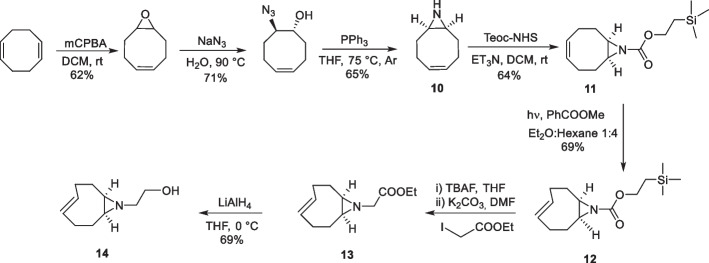
Synthetic scheme of aza-TCO

To preserve the fluorogenic characteristics of the reaction while allowing the attachment of a functional handle, Siegl et al. (2018) designed a *cis*-fused aziridine ring to *trans*-cyclooctene (aza-TCO). Computational studies confirmed that this fusion resulted in the desired “half-chair” conformation of TCO, enhancing the reaction rate. Further conformational analysis of the 4,5-dihydropyridazine intermediate showed a bridgehead hydrogen-aziridine nitrogen at a distance of 2.29 Å in its low-energy conformer, facilitating the hydrogen migration to yield a fluorescent product. Encouraged by the computational results aza-TCO was synthesized (Scheme 9) (Siegl et al. 2018). The synthesis involved the ring opening of the epoxide, yielding an azido alcohol which was subsequently converted into the aziridine (10) through Staudinger reduction-cyclization, resulting in a *cis*-ring fusion. Protection of the aziridine nitrogen with trimethylsilylethoxycarbonyl was found to be crucial for photoisomerization. After further deprotection and alkylation of the aziridine nitrogen, ethyl ester (13) was obtained, which was reduced to aza-TCO-alcohol (14).

As anticipated, the IEDDA products of aza-TCO (14) with tetrazines yielded fluorescent 1,4-dihydropyridazine products. The click products exhibited a large Stokes shift and tunable emission maxima depending on the substitution of tetrazine. Furthermore, 20–70-fold fluorescence enhancement was observed upon the reaction. Although the reported fluorescence quantum yield was relatively low, the authors claimed that it was sufficient for bioimaging applications. When reacted with diphenyl-s-tetrazine, aza-TCO exhibited a reaction rate that was 20 times faster (k_2_ = (0.7 ± 0.02) ×  10^3^ M^−1^s^−1^ compared of the axial diastereomer of TCO-OH (k_2_ = (0.04 ± 0.003) ×  10^3^ M^−1^s^−1^ and 1.5 times faster than that of d-TCO ((0.5 ± 0.008) ×  10^3^ M^−1^s^−1^, while it was slower than that of s-TCO (2 ± 0.02) ×  10^3^ M^−1^s^−1^. Additionally, aza-TCO showed good stability in the CD_3_OD-*d*_4_ solution at room temperature. Similar to other TCOs, exposure to thiol-containing groups led to a 1:1 mixture of *cis* and *trans* isomers within seven days in deuterated PBS (Siegl et al. 2018).

In the same study, an activated ester derivative of aza-TCO was effectively employed for fluorescent labeling of proteins. Moreover, aza-TCO-modified D-amino acids were effectively incorporated into the peptidoglycans of live bacteria after a 2-h incubation. A weak fluorescence signal persisted on the bacterial cell surface even after a 12 h incubation period with aza-TCO, indicating that a fraction of aza-TCO remained in its reactive *trans*-conformation.

#### Oxazolone fused-TCO (Ox-TCO)

**Scheme 10:**
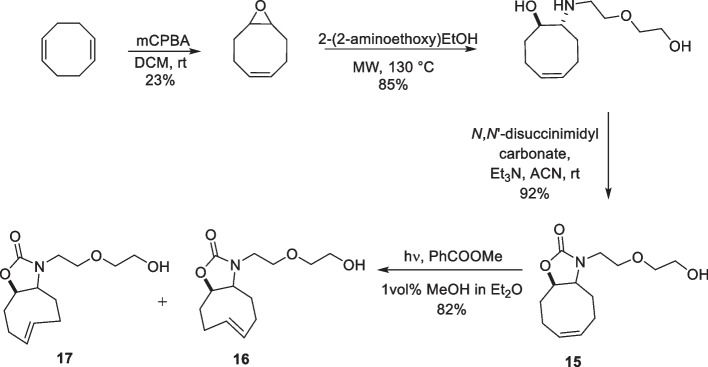
Synthetic scheme of Ox-TCO

Oxazolone-fused TCO was first reported in a biological assay by Kozma et al*. *(2016) as a hydrophilic ncAA. Dienophile-modified ncAAs are often hydrophobic, leading to nonspecific uptake (Nikic et al. 2014; Lang et al. 2012; Uttamapinant et al. 2015). To achieve easy and faster washout of reactive ncAAs from cells, a TCO with exocyclic heteroatoms was developed. The incorporation of a heteroatom increases the hydrophilicity while preserving the reactivity of TCOs (Jendralla 1983; Sletten and Bertozzi 2008). The synthesis was initiated by microwaving (*Z*)-9-oxabicyclo[6.1.0]non-4-ene with 2-(2-aminoethoxy)ethanol at 130 °C. The intermediate was subsequently reacted with *N,N*-disuccinimidyl carbonate, yielding a fused oxazolidine ring (15). The photoisomerization of the fused oxazolidine resulted in a mixture of two *trans*-isomers in a 4:1 ratio (Scheme 10). Attempts to isolate isomers (16 and 17) remained unsuccessful. Additionally, a long isomerization time (80 h) was required to obtain the desired product, which complicates the synthesis because silver leeching is observed over time (Hilderbrand et al. 2015). Although the synthesis of oxazolone-fused TCOs and their ring isomers has been attempted by a few groups, often low yields and fast compound degradation have been reported (Royzen et al. 2008; Darko et al. 2018). When reacted with benzylamino tetrazine, a 4:1 mixture of Ox-TCO isomers showed a k_2_ of (29 ± 0.6) ×  10^3^ M^−1^s^−1^ which is in the same range as *the trans*-cyclooct-4-enol reactivity (26 × 10^3^ M^−1^s^−1^. Ox-TCO is stable when stored in D_2_O at room temperature. Ox-TCO showed no degradation for up to 16 h in the presence of cysteine, showing a stability similar to that of TCO-OH (Hilderbrand et al. 2015). Unfortunately, no genetic incorporation of lysine-conjugated ox-TCO was observed when incubated in bacterial cell culture media. It was speculated that linker attachment at the five-membered oxazolidine ring could have led to limited recognition of ncAA by the bacterial synthetase (Kozma et al. 2016). In another study, hydrophilic Ox-TCO was used to study the *turn-on* fluorescence of Tz-quenched phenoxazine fluorogenic labels (Knorr et al. 2016).

#### 6-dioxo-TCO (DO-TCO)

Effects of endocyclic oxygen on the reactivity of TCO was first studied by Jendrella (1983), where 4,6-dioxo-TCO displayed a reaction rate that was 20–1000 times faster than TCO in cycloadditions with diphenylketene, cyclopentadiene, and 2,3-dimethyl butadiene. Studies by Sletten and Bertozzi (2008) also showed that the introduction of heteroatoms on cyclooctynes led to improved kinetics as well as increased hydrophilicity. Later on, in silico investigations by Gold et al. (2013) showed that the fast reactivity displayed by 3-oxocyclooctynes was partially attributed to the hyperconjugation effect of allylic oxygens. Heterocyclic **4,6-dioxo-TCO** was initially synthesized by Jendralla (1983, 2006) To enhance the washout times in fluorescence imaging, Kozma et al. introduced a modified version of DO-TCO for imaging of cell surface or cytosolic proteins via genetic incorporation. Notably, DO-TCO-Lys showed a remarkably fast washout from cytoplasm within 5 min, allowing labeling with fluorophore-conjugated Tz without any additional washing procedures (Kozma et al. 2016).

**Scheme 11:**
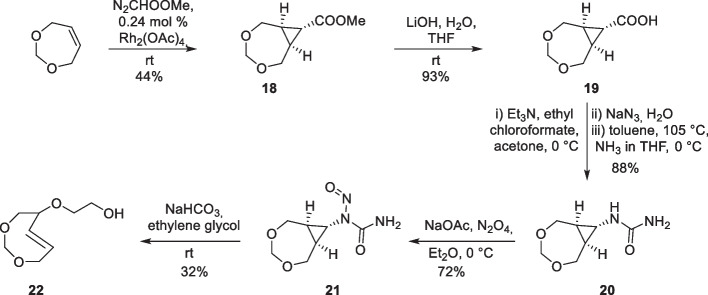
Synthesis of 4,6-dioxo-TCO (DO-TCO)

The synthesis of the 4,6-dioxo-TCO derivative was based on the method established by Jendrella with the addition of an extra glycol handle for further conjugation. The synthesis was based on stereoconversion and started with dioxepin, which was reacted with methyl diazoacetate in the presence of catalytic Rh_2_(OAc)_4_ to yield cyclopropyl carboxylate (18). This intermediate is subsequently hydrolyzed and transformed into urea (20) using Curtius rearrangement. Nitrosation of urea is then followed by the ring opening to afford the dioxo-TCO (22, Scheme 11) (Hilderbrand et al. 2015).

When reacted with benzylamino tetrazine in PBS at 37 °C, DO-TCO showed a second-order rate constant of k_2_ = (0.3 ± 0.003) ×  10^3^ M^−1^s^−1^. Notably, this value is significantly lower than the rate constant for TCO-OH ((33 ± 0.3) ×  10^3^ M^−1^s^−1^. Despite the anticipated higher internal strain in DO-TCO, the authors postulated that the decreased reactivity could be attributed to the additional steric hindrance caused by the glycol moiety positioned close to the *trans* double bond. Remarkably, when stored at -20 °C, no degradation or isomerization of DO-TCO was observed for up to six months. Slow isomerization of the compound was observed over 5 days at 37 °C in D_2_O. In the presence of cysteine, DO-TCO isomerized rapidly to *cis*, with no *trans* isomers present after 6 h. However, in the presence of mercaptoethanol, isomerization occurred along with decomposition, with about 70% remaining at a 25h time point (Hilderbrand et al. 2015).

#### *trans-*5-oxocene (oxo-TCO)

To expand the biorthogonal toolbox with small, hydrophilic, and reactive TCO derivatives, Lambert et al. (2017) designed two oxygen-containing isomers, namely *trans*-5-oxocene and *trans*-4-oxocene dienophiles. Computational predictions indicated that the presence of short C–O bonds in the eight-membered ring increases the strain on the double bond. This was confirmed by the calculated C–C=C–C dihedral angles for *the trans*-5-oxocene (134.6°) and *trans*-4-oxocene (134.4°), which were significantly shorter than those for the TCO (137.7°). Additionally, the energy barriers for the reaction with 3,6-diphenyl-s-tetrazine were significantly lower for *trans*-5-oxocene (ΔΔE‡ –1.23 kcal/mol, ΔE‡(ZPE) –1.54 kcal/mol, and ΔH‡ –1.44 kcal/mol) relative to *trans*-cyclooct-4-enol, while *trans*-4-oxocene exhibited similar energy barriers to *trans*-cyclooct-4-enol. These transition-state calculations suggest that *the trans*-5-oxocene is more reactive than *the trans*-4-oxocene. The higher ring strain in *trans*-4-oxocene was counteracted by the electron-withdrawing effect of the allylic oxygen, which deactivated the alkene through hyperconjugation (Lambert et al. 2017).

**Scheme 12:**
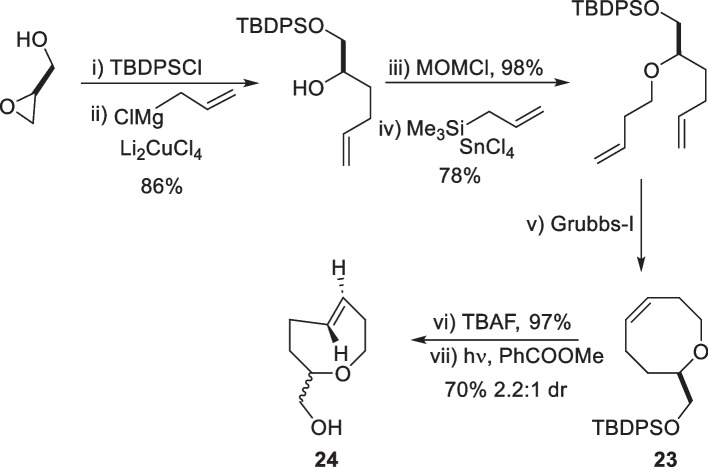
Synthesis of trans-5-oxocene

Alcohol-functionalized *trans*-5-oxocene (24) was synthesized through a series of allylation steps on glycidol to obtain a butenyl ether, followed by ring-closing metathesis. Photoisomerization of *cis*-oxocene (23) resulted in a 2.2:1 diastereomeric mixture of *trans*-oxocenes with the equatorial isomer as the major product (Scheme 12; 24). Notably, the separation of these diastereomers using silica gel chromatography was unsuccessful and preparative supercritical fluid chromatography was challenging, leading to the use of the diastereomeric mixture in subsequent experiments.

When reacted with 3,6-dipyridyl-s-tetrazine, equatorial oxo-TCO exhibited a rate constant of (44.1 ± 3) ×  10^3^ M^–1^ s^–1^ in PBS at 25 °C. As the isolation of a diastereomerically pure axial diastereomer was not successful, the rate constant was calculated to be 310 × 10^3^ M^–1^ s^–1^ based on the observed rates of the diastereomeric mixture and pure equatorial isomer, which was sevenfold more reactive than the equatorial diastereomer. The diastereomeric mixture of oxo-TCO showed fast kinetics when reacted with a succinic acid derivative of 3,6-dipyridyl-s-tetrazine with a second-order rate constant (k_2_) of (94.6 ± 6) ×  10^3^ M^–1^ s^–1^ in PBS at 25 °C. Although faster than the *trans*-cyclooct-4-enol isomers, the reaction remained approximately fourfold slower than conformationally strained d-TCO under similar reaction conditions (366 × 10^3^ M^–1^ s^–1^).

The logP value was experimentally determined (logP 0.51), indicating improved hydrophilicity compared to equatorial *trans*-cyclooct-4-enol (logP 1.11) and d-TCO (logP 0.94) (Darko et al. 2014). The major equatorial diastereomer of *trans*-5-oxocene showed less than 10% degradation in deuterated PBS after 1 week. In contrast, the more reactive axial (minor) diastereomer was prone to degradation, with a half-life of 36 h in PBS and complete degradation after 9 days. The compound exhibited higher isomerization in the presence of mercaptoethanol in buffered solutions (92%) than in organic solvents (8%). Cell imaging with a green fluorescent protein encoded Tz containing ncAAs and oxo-TCO resulted in rapid and quantitative labeling both in solution and in bacterial cells.

#### Other heteroatoms containing TCOs

**Fig. 6:**
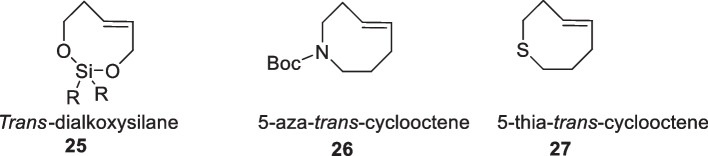
Structures of endocyclic heteroatom-containing TCOs

Other endocyclic heteroatom-containing *trans-*cyclooctenes have also been described. Toomoka reported the synthesis of *trans*-dialkoxysilane from (*E*)-2-pentene-1,5-diol and commercially available dichlorosilanes in the presence of AgNO_3_, yielding an eight-membered ring, followed by photoisomerization. In an alternative approach, ring-closing metathesis in the presence of Grubbs catalyst also yielded the ring. The obtained alkene was reported to display high reactivity in Diels–Alder reactions (Tomooka et al. 2014). Similarly, Woerpel also described a diastereoselective synthetic route for functionalized *trans*-dioxasilacyclooctenes through the insertion of silyenes into vinyl epoxides followed by the subsequent allylations of aldehydes (Prevost and Woerpel 2009). Finally, sulfur and nitrogen-containing TCOs have also been described but are mostly studied in the fields of transannular cyclization (Fig. 6) (Cerè et al. 1998; Royzen et al. 2011).

### TCO-Tetrazine – click-to-release reaction and mechanism

**Scheme 13:**
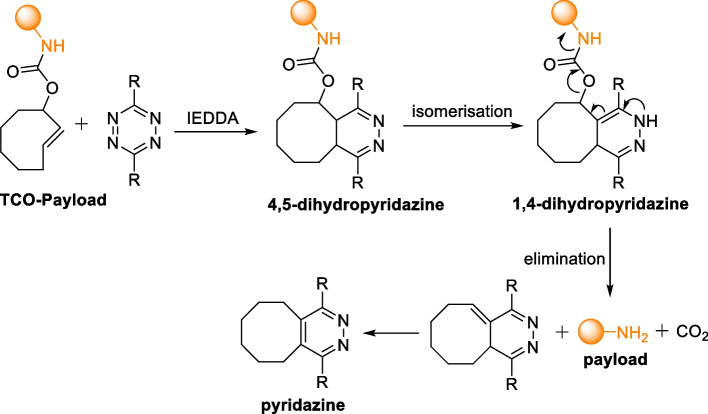
IEDDA decaging reaction between a TCO-carbamate conjugated to a drug molecule and tetrazine

The click-to-release strategy was first introduced by the Robillard group (Versteegen et al. 2013). The IEDDA click-to-release reaction involves two steps: first, cycloaddition occurs, followed by the elimination of a leaving group from the vinyl position of a TCO after the tautomerization of 4,5-dihydropyridazine and oxidation to pyridazine (Scheme 13).

In their initial study, Robillard et al. applied the strategy to activate a prodrug (TCO-carbamate-Doxorubicin) in vitro. When reacted with 3,6-dipyridyl-s-tetrazine, the axial diastereomer of the TCO reacts 16 times faster (k_2_ = 57.7 M^−1^ s^−1^ in ACN) than the equatorial diastereomer (k_2_ = 0.37 M^−1^ s^−1^). Despite the fast cycloaddition rate, only a 7% release of free doxorubicin (Dox) was observed after 3 h from the TCO-conjugated prodrug. However, when the prodrug was reacted with dimethyl tetrazine (DMT), a significantly faster release rate was observed. Specifically, 79% of the prodrug was released within 16 min, even though the cycloaddition step was slower, suggesting that a faster cycloaddition rate may not be the determining factor for elimination. In vitro experiments involving the caged prodrug (EC_50_ = 3.834 μM) demonstrated a notable reduction in toxicity in A431 tumor cells compared to free Dox (EC_50_ = 0.037 μM). Furthermore, the administration of the prodrug in combination DMT resulted in an almost complete restoration of Dox cytotoxicity (EC_50_ = 0.049 μM) (Versteegen et al. 2013).

**Fig. 7:**
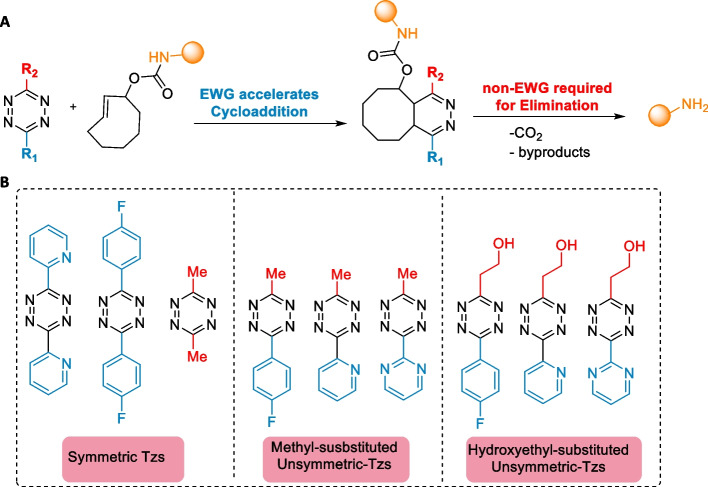
**a** Unsymmetric Tzs containing EWG and a non-EWG promote the elimination efficiency. **b** The release efficiencies of the symmetric and unsymmetric tetrazines in increasing order. Unsymmetric tetrazines Tzs exhibit superior release efficiency compared to their symmetric counterparts. Hydroxyethyl-substituted Tzs showed a higher release efficiency than methyl-substituted Tzs. Adapted with permission from Fan et al. (2016) Copyright 2016 Wiley–VCH Verlag GmbH & Co. KGaA, Weinheim

As the initial studies showed that a slower reacting DMT led to higher drug release when compared to faster reacting 3,6-dipyridyl-s-tetrazine, Chen and coworkers conducted a comprehensive investigation on the impact of Tz substituents on the elimination rates. Their findings revealed a significant release enhancement for asymmetric Tz compared with their symmetric counterparts. Notably, the presence of EWGs was found to promote the cycloaddition rate but impede elimination because of the formation of a stable intermediate after cycloaddition (Fig. 7). Tz incorporating a single EWG along with a small and relatively electron-neutral group exhibited an impressive release of up to 90% within 4 min in living cells (Fan et al. 2016).

**Scheme 14:**
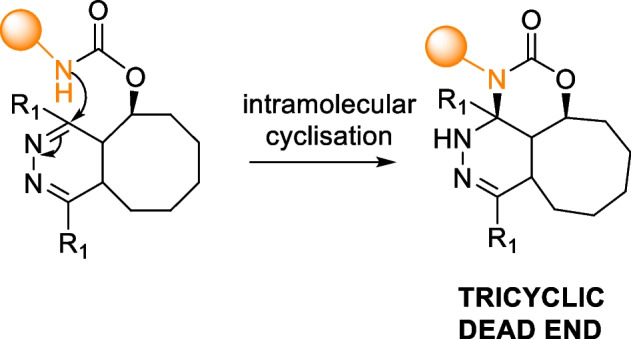
Formation of tricyclic dead-end product via intramolecular cyclization of carbamate amine onto tetrazine, resulting in an incomplete release

The TCO-Tz click-to-release reaction often leads to incomplete payload release. Studies by the Weissleder group revealed that the incomplete release was due to a dead-end tricyclic product resulting from the cyclization of the carbamate amine onto Tz after the cycloaddition (Scheme 14). To circumvent this pathway, the use of tertiary amines was proposed. However, as the amine linker originates from the drug, it may not always be the most desirable modification. Furthermore, the investigations revealed that the release is influenced by pH, with faster release rates observed under acidic conditions. The introduction of carboxylic acid-functionalized tetrazines, which act as proton donors to accelerate the tautomerization rate, has been proposed as an alternative approach to reducing the pH of the reaction medium. Notably, this approach resulted in the complete release as rapid tautomerization disfavors the dead-end cyclization product (Carlson et al. 2018).

**Scheme 15:**
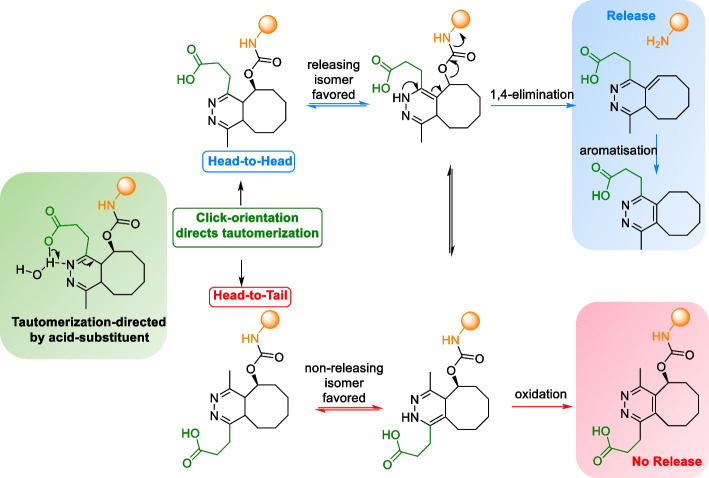
Tautomerization enhancement by acid-functionalized tetrazines via acid-assisted H transfer. The click orientation of the substituted tetrazine determines the pH-dependent drug release. A Head-to-Head orientation favors the formation of a releasing isomer, whereas a head-to-tail orientation favored the formation of a non-releasing isomer

When reacting with functionalized TCOs, asymmetric Tzs exhibit two possible orientations: head-to-head or head-to-tail. This study demonstrated that the reaction with head-to-head oriented Tzs displayed a preference for forming the releasing isomer, which was facilitated by assisted H-transfer from the vicinal carboxylic acid. Conversely, the head-to-tail orientation favors the formation of the non-releasing isomer (Scheme 15). (Carlson et al. 2018)

Sarris et al. introduced a novel class of Tz functionalized with amino ethyl groups, which facilitated the pH-independent and rapid release of the payload. The amino-ethyl group acts as an intramolecular proton donor as it is protonated at or below the physiological pH range, thereby accelerating the tautomerization of the initial 4,5 dihydro tautomer to the 1,4 dihydro tautomer. Additionally, the close proximity of the cationic ammonium group to carbamate also catalyzes the elimination reaction via H-transfer. The incorporation of cationic ammonium functionality resulted in a fourfold increase in the rate of elimination compared to the carboxylic acid group, which could be further enhanced by introducing EWGs onto the Tz scaffold (Sarris et al. 2018).

Later, mechanistic investigations conducted by Versteegen et al. (2018) revealed that the formation of a 1,4-dihydropyridazine tautomer was the key releasing factor. The 1,4-dihydropyridazine tautomer promptly liberates CO_2_ and amine upon its formation. Consequently, the rate of 1,4-dihydropyridazine formation is the rate limiting factor which determines the overall yield of the elimination reaction. In contrast, the 2,5-dihydropyridazine tautomer was slowly oxidized to pyridazine without liberating the payload, resulting in a lower elimination yield (< 40%). The research group also expanded the scope of release with other functional groups, such as aromatic carbonates, aliphatic and aromatic esters, as well as aliphatic, aromatic, and benzylic ethers (Versteegen et al. 2018). However, it should be noted that esters and carbonates are not stable in plasma or cellular environments, rendering them unsuitable for biological applications. Similarly, Davies et al. (2019a; b) also reported the release of alcohols and carboxylic acids from a TCO-carbamate conjugate employing a self-immolative benzyl ether linker.

**Scheme 16:**
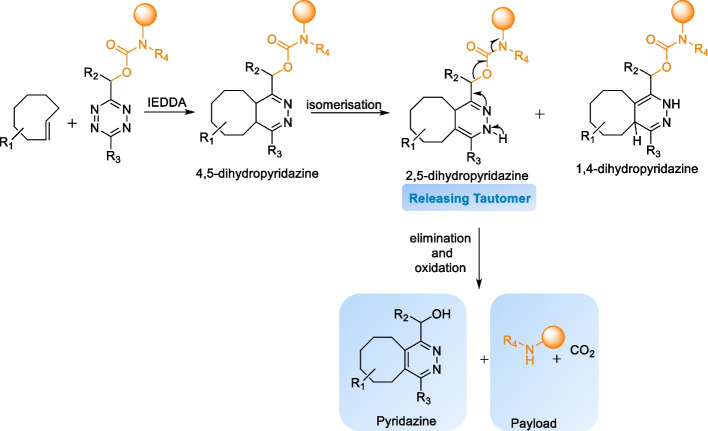
Schematic representation of TCO-triggered release of payload from tetrazine. The initially formed 4,5-DHP tautomerizes into 1,4-DHP and 2,5-DHP, of which 2,5 DHP liberates the amine-bound payload in conjunction with the formation of pyridazine Adapted from Onzen et al. (2020)with permission. Copyright © 2020, American Chemical Society

Robillard et al. also proposed a reverse strategy in which a prodrug is conjugated to a tetrazine, with TCO serving as the trigger for the elimination reaction. As the payload is released from Tz, allylic substitution on TCO is not required. Additionally, using TCO as a release trigger exposes it to in vivo conditions only for a shorter duration, and the stability requirements for TCO become less stringent. This allows for the use of more reactive TCOs as the trigger for release and enhances the reaction rate. The study demonstrated that, depending on the TCO used, an increase in reactivity (6–800-fold) and high elimination yields ranging from 67 to 93% were achieved through the 1,4-elimination of the carbamate substituted on a tetrazine. Mechanistic studies on the release from Tz based prodrug showed that 2,5-dihydropyridazine is the releasing isomer (Scheme 16).

Unlike Tz-triggered release, TCO-triggered elimination has only been demonstrated with secondary amines, indicating the limited scope of the leaving groups. This problem can be partially overcome by employing self-immolative linkers. However, further investigation is necessary to expand the chemical scope (Onzen et al. 2020). Additionally, release strategies typically require an excess trigger concentration to ensure complete release. However, the reverse approach allows for the use of lower trigger doses, as faster kinetics can be achieved using faster reacting TCOs. Moreover, the use of Tz-protecting groups for prodrugs offers greater flexibility in synthesis and conjugation than TCOs and shows greater stability under in vivo conditions. TCO-triggered cleavage of Tz-prodrugs holds significant potential for improving the cycloaddition kinetics as well as enhancing the stability of antibody–drug conjugates (ADCs) compared to TCO-conjugated ADCs.

Since the discovery of the click-to-release strategy, different derivatives of releasing TCOs have been developed. These reported compounds are discussed in the following sections.

#### *Trans*-cyclooct-2-en-1-yl carbamate (r-TCO)

**Scheme 17:**
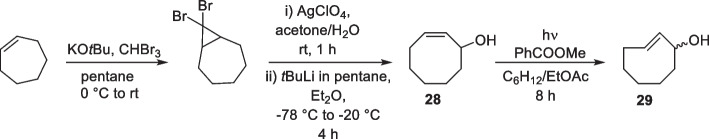
Synthesis of r-TCO

r-TCO contains an allylic carbamate substituent for payload release. Upon the reaction of r-TCO with Tz, post-click tautomerization occurs, resulting in 1,4-elimination with cleavage of the carbamate and subsequent elimination of CO_2_ and free amine (Versteegen et al. 2013). The synthesis of r-TCO involves the initial epoxidation of cycloheptene, followed by ring expansion to obtain *cis*-cyclooct-2-enol (28). This compound is then subjected to photoisomerization, resulting in the formation of two isomers of *trans*-cyclooct-2-enol (29; Scheme 17) that can be further derivatized (Nikic et al. 2014). Notably, the allylic substituent leads to a 20-fold decrease in reactivity of the axial r-TCO (k_2_ = 57.7 M^−1^s^−1^ when compared to *trans*-cyclooct-4-enol (k_2_ = 1140 M^−1^s^−1^ in ACN at 20 °C (Versteegen et al. 2013). An unexpectedly larger difference in reactivity, with 156-fold lower reactivity was measured for the equatorial hydroxyl-derived carbamate when compared to the axial substitution. This discrepancy in reactivity was attributed to steric hindrance as well as potential electronic effects. With a half-life of 20 days, r-TCO showed comparable stability to the *trans*-cyclooct-4-enol derivatives commonly used for IEDDA-based click ligation. Notably, r-TCO contains only one functional group, which allows chemical modification. Nevertheless, it has found utility as a click-removable tag, frequently employed for the release of cytotoxic drugs, enzymes, and proteins, and nanoparticle-based prodrug activation (Mejia Oneto et al. 2016; Zhang et al. 2016; Li et al. 2014).

#### Click-cleavable TCO (cTCO)

To develop a bioorthogonally activatable ADC, Robillard et al. designed a click-cleavable TCO with an additional site for functionalization. This enabled chemically controlled pretargeted drug release from an ADC caged by c-TCO following an in vivo reaction between a Tz and cTCO-caged ADC (Rossin et al. 2016). The introduction of an additional functional group on the eight-membered ring offered the possibility of fine-tuning the pharmacokinetics of TCO by attaching a hydrophilic moiety. This approach has been employed to incorporate polar group on the TCO-core to improve the solubility and hydrophilicity of TCO-caged prodrugs (Mejia Oneto et al. 2016; Wu et al. 2021).

**Scheme 18:**
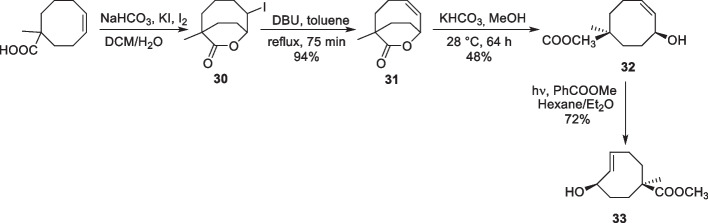
Synthesis of cTCO

The synthesis of cTCO (33) involved a lengthy 9-step procedure, starting with the installation of a carboxylic acid and a methyl group on 1,4-cyclooctadiene. Iodolactonization followed by hydrogen iodide elimination and lactone hydrolysis was crucial for stereoselective introduction of the allylic hydroxyl group in *the cis* configuration with the COOH moiety. The presence of an α-methyl group prevented undesired epimerization during the lactone hydrolysis step (Scheme 18). Although the synthetic pathway allows for stereospecific introduction of an additional functional group, the procedure requires considerable effort. cTCO exhibited an exceptionally long shelf life, with a half-life of 2.6 years for the *trans* isomer in a PBS stock solution at 4 °C. In serum, the *trans* isomer demonstrated a stability half-life of 5 days, showing only a decrease in reactivity towards Tz without any payload release (Rossin et al. 2016). The bifunctional cTCO has found many applications in drug release, which is discussed in the application section.

#### Cleavable C_2_-symmetric *trans*-Cyclooctene (C_2_TCO)

**Scheme 19:**
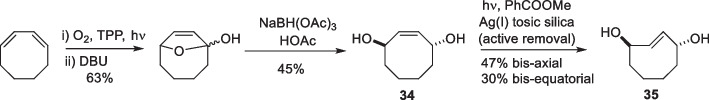
Synthesis of C_2_-TCO

Mikula et al. developed a C_2_-symmetric *trans*-cyclooctene (C_2_TCO; 35) with two allylic hydroxyl groups in an axial configuration. Starting with 1,3-cyclooctadiene, the desired C_2_TCO-diol (bis-axial) was prepared in three steps (Scheme 19). The synthesis involved photooxygenation of 1,3-cyclooctadiene, followed by the formation of a lactol intermediate. The reduction of the lactol afforded cyclooct-2-ene-1,4-diol (34). The flow-photoisomerization of bis-diol (34) using TAg silica gel yielded bis-axial-TCO (47%) and bis-equatorial-TCO (30%). When reacted with Tzs, second-order rate constants up to 400 M^–1^s^–1^ were measured, followed by an instantaneous release. Complete cleavage (> 99%) of the C_2_TCO conjugates was achieved within 3 min at low reactant concentrations. When bis sarcosinyl modified-C_2_TCO was incubated in cell media (10% FBS) at 37 °C, more than 97% of C_2_TCO remained stable for up to 48 h (Wilkovitsch et al. 2020).

C_2_TCO conjugated to mAb and AF594 fluorophore was applied in a bioorthogonal *turn-off* strategy for extracellular and intracellular cleavage of the fluorophore. Following the addition of 3-(1,2,4,5-tetrazin-3-yl)propanoic acid (HPA-Tz), rapid cleavage of the fluorophore and significant reduction in the fluorescence signal were observed within 6 min of Tz addition. Although fast and complete disassembly can be achieved with C_2_TCO, a selective release of one of the two groups is not possible, which could be an obstacle when the controlled delivery of molecular cargo is required (Wilkovitsch et al. 2020)..

#### Dioxolane-fused cleavable TCO (dcTCO)

**Scheme 20:**
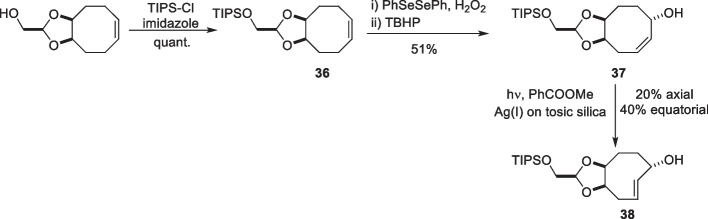
Synthesis of dcTCO

Mikula et al. also developed a synthetic approach to enhance the accessibility of release TCOs through oxidative desymmetrization of a symmetric precursor (Kuba et al. 2023). This approach limited the number of possible isomers produced compared to the allylic oxidation of non-symmetrical precursors, resulting in a mixture of regio-and stereoisomers, complicating further synthesis. The diastereoselective synthesis involved the oxidative desymmetrization of triisopropylsilyl (TIPS)-protected dioxolane-fused CCO (36) mediated by diphenyl diselenide. This resulted in a shift of the double bond relative to the fused dioxolane ring. Subsequent photoisomerization allowed the isolation of both equatorial and axial isomers of 38 (Scheme 20).

The altered position of the fused dioxolane ring from the 5’/6’ (d-TCO) to the 4’/5’ position (dcTCO) allowed the double bond to adopt a less strained crown conformation, thereby increasing the stability while decreasing the reactivity compared to d-TCO. When reacted with DMT, the axial dcTCO derivative showed similar reactivity to r-TCO-PEG_4_, with k_2_ values of 74 M^−1^s^−1^ and 80 M^−1^s^−1^, respectively. In contrast, equatorial dcTCO exhibited a considerably lower reactivity, with a 30-fold decrease in reactivity compared to that of the axial isomer. This decrease in reactivity was attributed to the increased steric hindrance posed by the carbamate group at the equatorial position.

Incubation of axial dcTCO in PBS at 37 °C showed good stability, with 99% of the dcTCO remaining intact after 96 h, surpassing the high stability of rTCO. Additionally, logP calculations for a range of derivatives indicated a significantly increased hydrophilicity of dcTCO derivatives compared to r-TCO with an average difference in clog P_7.4_ value of − 1.74. Cell experiments demonstrated a > 1000-fold reduction in cytotoxicity of the dcTCO-conjugated prodrug of combretastatin A4 (CA4) compared to native CA4. The addition of Tz led a complete restoration of activity of the prodrug following the Tz-triggered cleavage of the dcTCO linker (Kuba et al. 2023).

#### Cleavable s-TCO

**Scheme 21:**
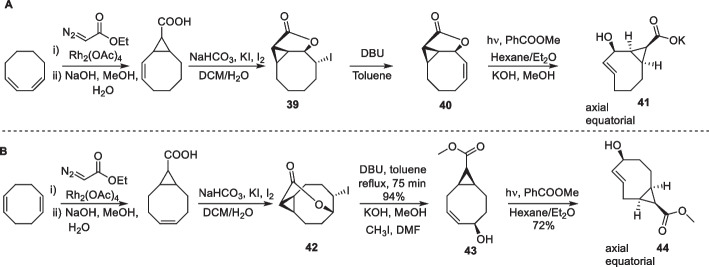
**a**: Synthesis of difunctionalized s-TCO derivative starting from 1,3-cyclooctadiene. **b** Synthesis of difunctionalized s-TCO derivative starting from 1,5-cyclooctadiene

The reactivity of release TCOs with allylic substitution is lower than that of the parent TCO, necessitating a higher dose of the tetrazine activator for efficient release. An increase in the reaction rate is desirable in vivo as it enables the use of a lower activator dose (Versteegen et al. 2013). Based on the reactivity enhancement of s-TCO through *cis*-fusion of a cyclopropane ring, Robillard et al. envisioned the development of a cleavable s-TCO with a carboxylic acid substituent at the *syn* position on the fused cyclopropane ring. Two different approaches were described, starting from 1,3-cyclooctadiene and 1,5-cyclooctadiene (Schemes 21 A and B). Similar to the synthesis of bifunctional c-TCO, halolactonization, hydrogen iodide elimination, and lactone hydrolysis allowed the installation of allylic hydroxyl and carboxylic substituents. Both approaches resulted in the formation of mixture of axial and equatorial isomers of *trans*-cyclooctene (Liu et al. 2023).

When studied for reactivity, the equatorial isomers of both s-TCOs were slower than their axial isomers. The s-TCO cleavable linkers derivatized with *N*-methyl benzylamine demonstrated higher reactivity with DMT in ACN when compared to the parent r-TCO (0.54 M^−1^s^−1^ with similar allylic benzylamine substituent (Versteegen et al. 2013; Rossin et al. 2016). 1,3-s-TCO (41) showed a 15-fold increase in rate with a k_2_ of 8.0 M^−1^s^−1^, whereas 1,5-s-TCO (44) gained a fivefold increase in reactivity with a k_2_ of 2.9 M^−1^s^−1^ (Rossin et al. 2016). In vitro studies investigating payload release upon treatment with DMT revealed a complete release within 30 min for both s-TCOs. The stability evaluation of s-TCO-CC49 conjugates in PBS at 4 °C revealed excellent stability for up to one year for 1,5-sTCO (44). In contrast, 1,3-s-TCO (41) was deactivated within a few days, rendering it unsuitable for release applications. In vivo, 1,5-s-TCO showed a half-life of 5.6 days, which is similar to that of the state-of-art TCO linkers with similar allylic benzylamine substituent (Rossin et al. 2016, 2018).

**Scheme 22:**
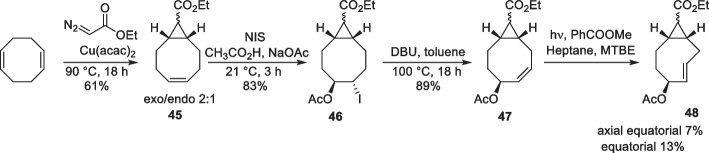
Synthesis of difunctionalized s-TCO derivative starting from 1,5-cyclooctadiene

Similarly, Rutjes et al. developed a synthetic pathway to obtain strained difunctionalized releasing TCO. The novel synthetic pathway allowed for the installation of the two functional groups in only four steps (Sondag et al. 2023). The monocyclopropanation of 1,5-cyclooctadiene with ethyl diazoacetate afforded a mixture of the *exo* and *endo*-diastereomers in a 2:1 ratio, respectively. The key step involved the activation of *exo*- and *endo*-diastereomers by *N*-iodosuccinimide (NIS) in the presence of sodium acetate and acetic acid, generating acetylated iodohydrin (46) in a single-step (Okamoto et al. 1987). Subsequent dehydroiodination reaction resulted in difunctionalized *cis*-cyclooctene (47), which was photoisomerized into the TCO derivative, yielding both axial and equatorial isomers (48; Scheme 22). In contrast to the diastereomers formed through the *exo*-pathway, those formed through the *endo*-pathway remained inseparable.

The click reaction of axial *exo*-TCO demonstrated k_2_ values of 594.0 M^−1^s^−1^ and 0.859 M^−1^s^−1^ with 3,6-dipyridyl-s tetrazine and 3,6-dimethyl-1,2,4,5-tetrazine, respectively, in ACN at room temperature. In vitro experiments showed that over 50% of the payload was released when the *exo*-axial TCO reacted with the DMT. When assessed for stability, the free carboxylic acid derivative of axial TCO remained stable after seven days in PBS and up to 4h in mouse serum at 37 °C. Although no isomerization was observed, the compound degraded over time (16h) in mouse serum (Sondag et al. 2023).

The *exo*-derivative of the difunctionalized cleavable s-TCO linker was modified with Dox at the allylic alcohol. When evaluated in HeLa cells, s-TCO-caged Dox showed a 21-fold increase in cytotoxicity following the addition of DMT compared with the prodrug alone. The improved synthetic pathways and kinetics of these release TCOs open possibilities for further fine-tuning of the parameters of the in vivo cleavage reactions.

### Applications of *trans*-cyclooctenes in nuclear medicine

Nuclear imaging techniques use radiolabeled biological vectors or small molecules known as radiotracers to image targeted cellular functions or pathological processes using single-photon emission computed tomography (SPECT) or positron emission tomography (PET). The high sensitivity of nuclear imaging techniques allows for the visualization of the accumulation of radiotracers in biochemical processes at nanomolar to micromolar concentrations. The incorporation of TCO-Tz ligation in radiochemistry has increased over time owing to its rapid reaction kinetics and biocompatibility. In particular, TCOs have been used as a radiolabeling strategy or as an in vivo labeling method through a pretargeting strategy for imaging and therapy.

#### ^18^F-labeled TCO as prosthetic group for radiolabeling

The potential of TCO as a radioligand was initially investigated by Fox et al. in 2010, who sucessfully demonstrated that [^18^F]F-TCO reacted with 3,6-dipyridyl-s-tetrazine within seconds achieving a excellent radiochemical yield (RCY) of 98% at low micromolar concentrations (Li et al. 2010a). Fluorine-18-radiofluorination of TCO can be performed directly by nucleophilic substitution of tosylate precursors with [^18^F]fluoride. [^18^F]F-TCO was first used in vivo as a radiolabeling method for a Tz-conjugated RGD (Arg-Gly-Asp) peptide for PET imaging of α_v_β_3_ integrins. Labeling of the RGD peptide with ^18^F using IEDDA was achieved in 5 min with efficient conjugation (RCY = 90%) at low concentrations of the Tz precursor molecule. PET imaging of U87MG xenografts, which express α_v_β_3_ integrins, showed a tracer accumulation in tumors with uptake values of 2.7 ± 0.5% ID/g. However, the pharmacokinetics of radiofluorinated tracers were suboptimal. The TCO-Tz ligation product exhibited considerable hydrophobicity, resulting in substantial uptake of activity in the liver and abdomen (Selvaraj et al. 2011). In subsequent studies, a bifunctional tetrazine-maleimide partner was introduced for site-specific labeling via cysteine residues on peptides and proteins. Here too, labeling with [^18^F]F-TCO allowed for high RCY > 95% and high specific activity of 111–222 GBq µmol^−1^ for ^18^F-TCO-Tz-cRGDyC peptide (Liu et al. 2013b).

Similarly, [^18^F]F-TCO-Tz ligation has been used for radiolabeling exendin-4, a glucagon-like peptide-1-receptor (GLP-1R) agonist for imaging islet transplantation models. Notably, the use of TCO-tetrazine ligation for radiolabeling allowed for a higher specific activity of the radiotracer when compared to the use of ^18^F-labeled prosthetic groups, such as *N*-2-([4-^18^F]fluorobenzamido)ethylmaleimide ([^18^F]FBEM) and ^18^F-fluorobenzaldehyde. High specific activity is crucial to prevent blocking effects when receptor expression is limited (Wu et al. 2013; Wang et al. 2012). Importantly, [^18^F]F-TCO, as a prosthetic group, enables rapid radiolabeling at room temperature and aqueous conditions, which are ideal for labeling peptides. However, the higher lipophilicity of the ligation product as well as the formation of dihydropyridazine isomers can potentially hamper clinical translation.

[^18^F]F-TCO has been successfully employed for the radiofluorination of small molecules such as AZD2281, a poly(ADP-ribose)polymerase 1 (PARP1) inhibitor without affecting the affinity of the compotowardsward its target (Keliher et al. 2011). The in vivo PET-imaging results demonstrated a specific accumulation of [^18^F]FAZD2281 in MDA-MB-436 tumor-bearing mice with PARP1 overexpression (Reiner et al. 2011).

In another study, Wang et al. (2016) successfully radiolabeled s-TCO with ^18^F via nucleophilic substitution of a tosylated precursor. The exceptionally fast reaction kinetics of [^18^F]F-sTCO allowed for the use of more stable diphenyl-s-tetrazine-conjugated peptides, resulting in improved in vivo stability of the radiolabeled peptides compared to those conjugated with 3,6-dipyridyl-s-tetrazine. [^18^F]F-sTCO was stable in PBS (pH 7.4) for up to 2 h at 37 °C. In fetal bovine serum, [^18^F]F-sTCO showed a 74% intact tracer after one hour of incubation. The lower radiochemical stability of s-TCO for PET probe assembly is acceptable, as the IEDDA reaction occurs within a few minutes. Furthermore, [^18^F]F-s-TCO-DiPhTz-RGDyK demonstrated persistent and prominent uptake in U87MG tumors, with tumor uptake values increasing up to 8.9 ± 0.5% ID/g at 4h p.i. This was an improvement compared to [^18^F]F-TCO-labeled 3,6-dipyridyl-s-tetrazine-RGD conjugates, where the tumor uptake decreased over time. The increased tumor uptake of [^18^F]F-s-TCO-DiPhTz-RGDyK at later time points suggested that it possessed prolonged circulation, which contributed to persistent tumor uptake of the labeled peptide. This observation was further translated by Wang et al. to improve tumor uptake of different radiolabeled peptides, such as NT20.3, Exendin-4, and bombesin, through enhanced blood circulation afforded by the introduction of the [^18^F]F-s-TCO-DiPhTz system in the above-mentioned peptides (Wang et al. 2020). The mechanism of enhanced blood circulation was attributed to the binding of the diphenyl tetrazine moiety with plasma proteins, specifically with hemopexin and transferrin. Generally, the lipophilicity of the radiolabeled ^18^F-labeled TCO-Tz ligation product results in modest tumor-to-background contrast. To improve the contrast ratios, novel radioligands were constructed using [^18^F]F-s-TCO and neurotensin conjugates of the more hydrophilic Tz (3,6-di(2-hydroxyethyl)tetrazine). This Tz showed fast kinetics (k_2_ = 11.5 × 10^3^ M^−1^s^−1^ and yielded a PET-imaging probe with less nonspecific uptake in the H1299 nonsmall cell lung carcinoma tumor model (Feng et al. 2020).

**Fig. 8:**
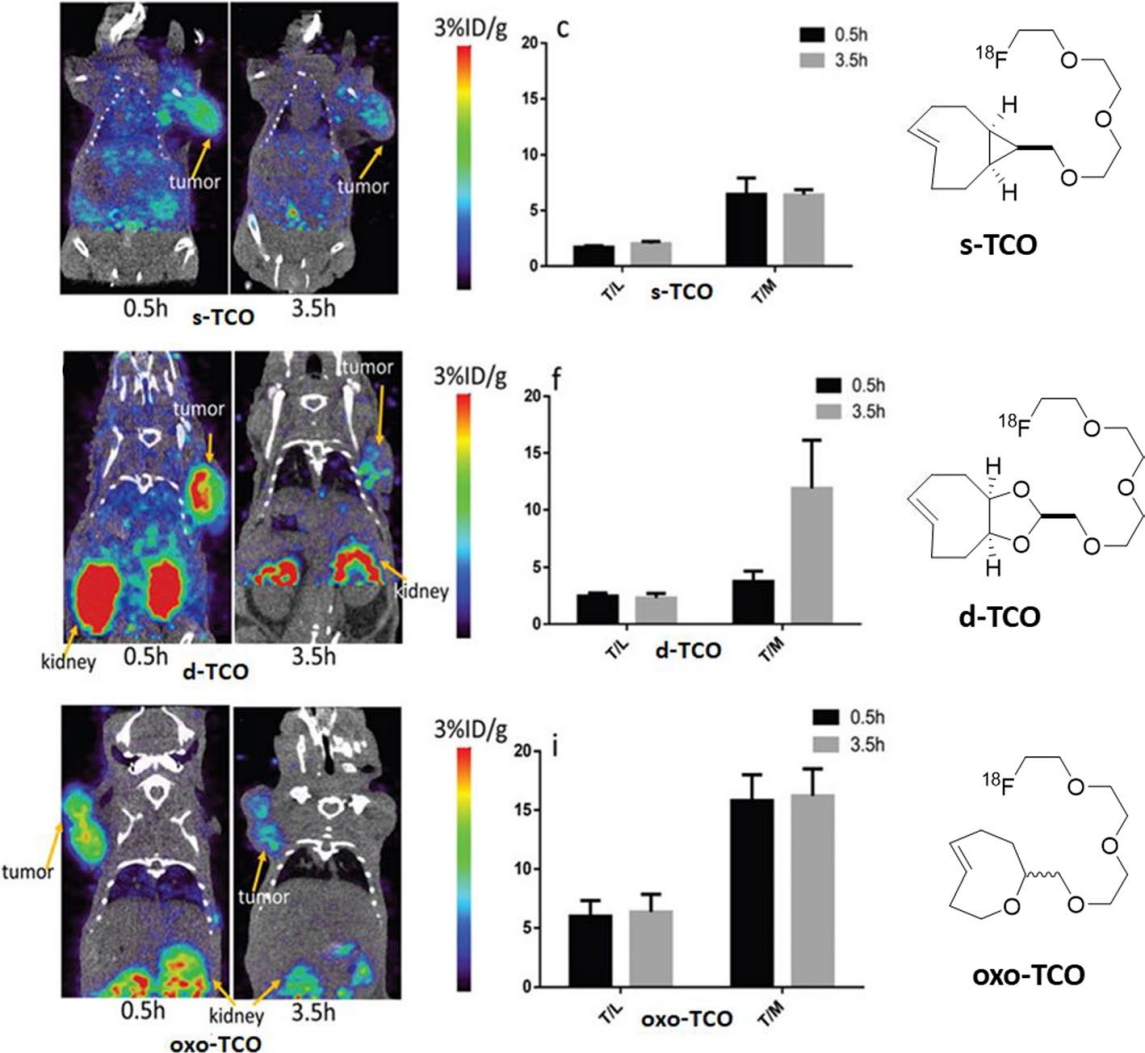
Representative PET/CT images of PC-3 tumor-bearing mice at 0.5h and 3.5h post-injection with [^18^F]F s-TCO, [^18^F]F d-TCO, and [^18^F]F-OxoTCO-derived NT-analogs. Image-derived tumor-to-liver (T/L) and tumor-to-muscle (T/M) ratios from the conversion of the region of interest to %ID/g. The grey and black bars represent the tumor-to-organ ratios at 0.5h and 3.5h respectively. The tumor-to-background ratio was highest with the oxoTCO-derived NT analog. Reproduced from Wang et al. (2019 with permission from the Royal Society of Chemistry)

Recently, Wang et al. developed ^18^F-labeled *trans*-5-oxocene TCO ([^18^F]F-oxoTCO), which exhibits enhanced hydrophilicity, enabling rapid clearance of the radiotracer from non-target organs. The tracer was 85% intact after 1h of incubation in PBS. Tracer degradation was attributed to radiolysis. In a comparison study, [^18^F]F-OxoTCO, [^18^F]F-d-TCO, and [^18^F]F-s-TCO were used as prosthetic groups for rapid ligation with the Tz-conjugated neurotensin peptide Lys-NT20.3. The tracers were evaluated for in vivo PET imaging in neurotensin receptor-positive PC-3 tumor-bearing mice. The [^18^F]F-OxoTCO radiolabeled peptide showed similar tumor uptake as [^18^F]F-s-TCO and [^18^F]F-d-TCO labeled peptide. However, the ^18^F-labeled oxoTCO petide resulted in a significantly higher tumor-to-muscle ratio of 15.8 ± 2.2, compared to [^18^F]F-s-TCO (6.5 ± 1.5) and [^18^F]F-d-TCO (3.8 ± 0.9) at 30 min p.i. The tumor-to-muscle ratio remained high after 3.5 h (16.2 ± 2.3), which was attributed to lower nonspecific uptake of the [^18^F]F-OxoTCO radiolabeled peptide due to its increased hydrophilicity (Fig. 8) (Wang et al. 2019).

#### TCO as a bioorthogonal tag for pretargeted imaging and therapy

Apart from being used as prosthetic groups, TCOs have also been widely used in pretargeted imaging as bioorthogonal tags in combination with labeled tetrazines. The pretargeting approach is a multistep procedure used in immuno-PET imaging and radioimmunotherapy (RIT) to overcome the slow clearance associated with directly radiolabeled antibodies. This is a two-step procedure. Initially, a tumor targeting antibody conjugated with a bioorthogonal tag is administered to accumulate at the target site. Subsequently, a radiolabeled molecule that specifically binds to the bioorthogonal tag is injected. The fraction of the radiolabeled molecule that is not bound to the tag is rapidly eliminated from the body. For imaging, this allows the use of short-lived radioisotopes decreasing the radiation burden while improving image contrast (Goodwin 1995). Whereas in PRIT, the fast clearance of the radiolabeled vector decreases the radiation burden to non-target organs. Despite previous investigations into other pretargeting methods, the IEDDA reaction between TCO and Tz has proven to be a highly promising approach because of its exceptionally fast reaction kinetics, with a rate constants measured up to 10^6−7^ M^−1^s^−1^ (Altai et al. 2017; Darko et al. 2014). Although both TCO and Tz can be radiolabeled, most pretargeting studies have utilized a TCO-conjugated mAb as the targeting vector and a Tz-based radioligand.

**Fig. 9:**
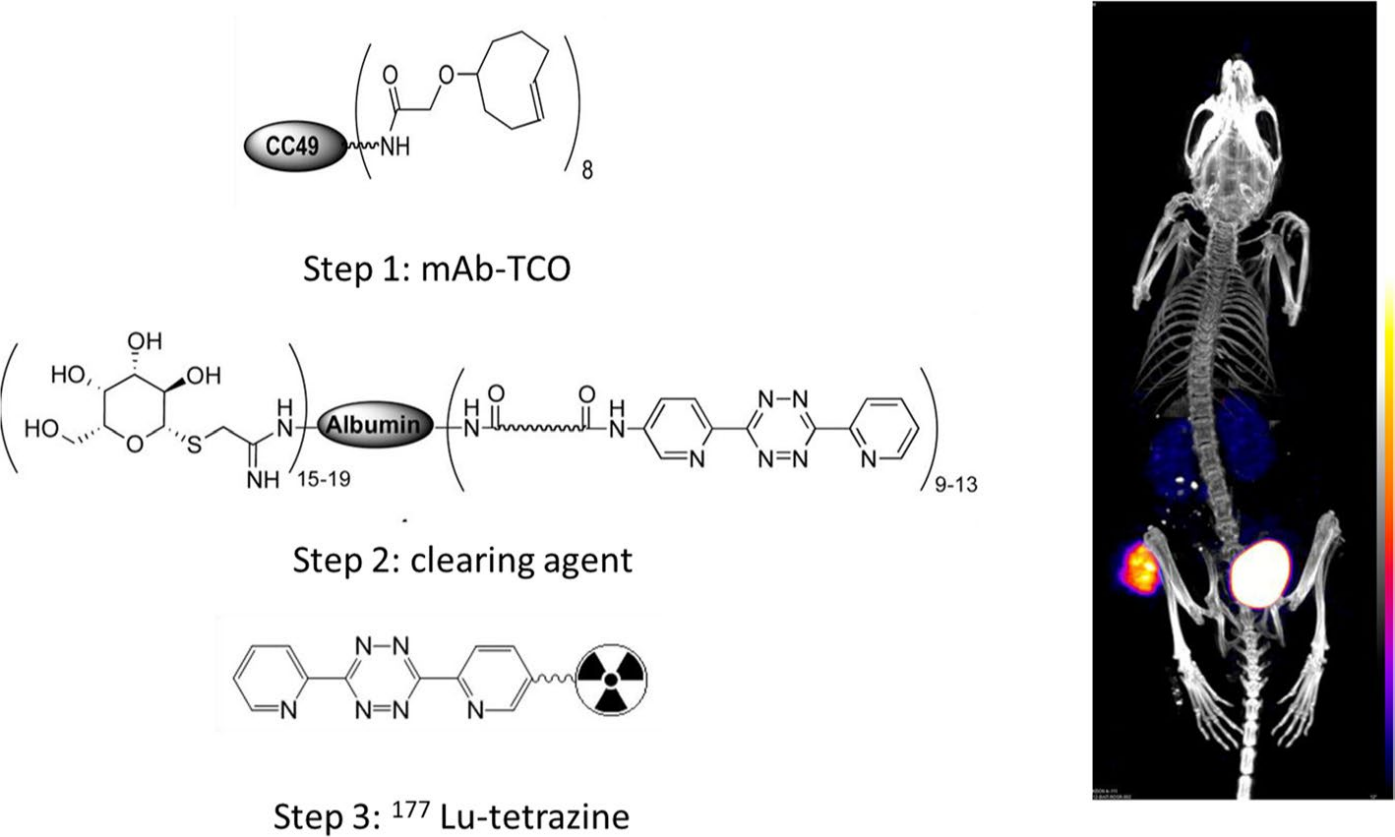
Representative SPECT/CT image of tumor-bearing mice injected with CC49-TCO, followed by two doses of galactose-albumin-tetrazine clearing agent (30 and 48h post-mAb injection) and ^177^Lu-labeled tetrazine at 50h post-mAb injection. The image shows high radioactivity uptake in the tumor and low uptake in non-target organs. Most of the activity is present in the bladder. Adapted from Rossin et al. (2014) with permission Copyright 2014 American Chemical Society

Pioneering work by Robillard et al. demonstrated the potential of TCO-Tz ligation as a pretargeting strategy for imaging tumors in vivo (Rossin et al. 2010). Here, a *trans*-cyclooct-4-enol-modified Ab conjugate (CC49-TCO) was used in combination with a ^111^In-labeled DOTA-Tz as a radioligand for the SPECT imaging of TAG-72 expressing colorectal cancer xenografts. This approach achieved excellent image contrast with a tumor-to-muscle ratio of 13:1 (Rossin et al. 2010). In subsequent studies, the use of the axial isomer of TCO with a short benzamide linker resulted in a tenfold increase in reactivity and 1.55-fold improvement in in vivo stability. This allowed for a longer lag time of 72h, between the mAb-TCO and radiolabeled Tz injection without compromising the tumor uptake while significantly increasing the tumor-to-blood ratio (Rossin et al. 2013a). To further enhance the pharmacokinetics of the mAb-TCO conjugate, the linker was modified to an acetamide, which further increased the stability of the conjugated TCO through greater steric hindrance around the TCO tag. Additionally, two doses of clearing agent consisting of albumin conjugated to both galactose and tetrazine was used to clear the non-bound CC49-TCO from the circulation. This resulted in improved image contrast with tumor accumulation of ^177^Lu-labeled DOTA-Tz up to 9.35% ID/g (Fig. 9) (Rossin et al. 2014).

Several other groups have since then adopted the strategy to visualize different tumor types with mAb conjugated to TCO in combination with radiolabeled Tzs (Cook et al. 2016; Meyer et al. 2016; Evans et al. 2014; Zeglis et al. 2013; Garcia et al. 2016). Additionally, the approach was also successfully applied to delineate tumors in a pancreatic cancer xenograft model, where high antigen circulation and target internalization complicate the in vivo targeting process (Houghton et al. 2016). Early efforts by Zeglis et al. already showed the superiority of the pretargeting approach based on TCO-Tz ligation when compared to directly radiolabeled antibodies. Although a higher absolute tumor uptake of 33% ID/g was observed for directly labeled [^64^Cu]Cu-NOTA-A33 in SW1222 xenografts, a lower effective dose (0.0124 mSv/MBq) was observed without compromising image contrast (Zeglis et al. 2013). It is noteworthy that a lower radiotracer uptake can be a disadvantage in a pretargeted therapy setting where a high absorbed dose in the tumor is required. Similar results were observed in a recent study using a TCO-conjugated anti-CD44v6 chimeric mAb U36 in combination with ^89^Zr-labeled Tz for imaging head and neck squamous cell carcinoma (HNSCC) xenografts. The pretargeted approach led to a lower absolute tumor uptake (1.5 ± 2% ID/g at 72 h p.i.) of the radiotracer compared to the directly labeled mAb (17.1 ± 3.0% ID/g at 72 h p.i.). However, the tumor-to-non-target tissue ratios obtained were comparable for both approach and a lower absorbed doses were observed for pretargeted approach (Lumen et al. 2022). With the recent advancements in direct Tz labeling procedures, further studies on pretargeting have been conducted with ^18^F-labeled Tzs in combination with mAb-TCO (Garcia-Vazquez et al. 2021; Keinanen et al. 2016, 2017; Steen et al. 2021). Although the absolute tumor uptake values are generally low with ^18^F-labeled Tzs, studies have shown significant improvements in the tumor-to-muscle ratios up to 20.8 resulting in images with better contrast while reducing the radiation burden (Battisti et al. 2021; Garcia-Vazquez et al. 2022).

The use of TCO-Tz ligation has attracted interest in the field of brain imaging. Recently, bispecific antibodies to the transferrin receptor (TfR) were conjugated with TCO, either for radiolabeling or pretargeted imaging across the blood–brain barrier (BBB). TfR is expressed in the endothelial cells of the BBB and neurons and facilitates the transport of antibodies through the BBB via receptor-mediated transcytosis (Hersom et al. 2016). Based on this, bispecific antibodies have been used for the visualization of β-amyloid (Aβ) plaques. Sehlin et al. reported the use of TCO-functionalized bispecific antibody ligands RmAb158-scFv8D3 and Tribody A2, which were subsequently labeled with [^18^F]F-Tzs. As the conjugates were pre-clicked before injection, the physical half-life of Fluorine-18 (t_1/2_ = 110 min) was too short to match the slow pharmacokinetics of the labeled antibody, resulting in no significant uptake differences between the wild-type and tg-ArcSwe mouse models, with increased Aβ aggregation and production (Syvanen et al. 2020). Recently, a pretargeting approach was applied using a brain-penetrating mAb31 (mAb31-BrainShuttle). TCO-functionalized mAb31 was used with tritium-labeled-Tz for pretargeted labeling of Aβ plaques in the PS2APP Alzheimer’s disease mouse model. A pre-clicked mAb-TCO-Tz conjugate successfully passed the BBB ex vivo and allowed for specific visualization of Aβ plaques. However, no significant labeling of Aβ plaques was observed in vivo (Bredack et al. 2022). In a proof-of-concept study, Shalgunov et al. demonstrated an in vivo click reaction of ^18^F-labeled Tzs with TCO in the brain. TCO-tagged PeptoBrush polymer was injected into the right striatum of Long-Evans rats followed by radiolabeled Tz, where the accumulation of Tz was visible at the point of Peptobrush injection (Shalgunov et al. 2023). However, the study still leaves a gap in imaging targets beyond the BBB, as the approach described was based on intracerebral injection of both click components.

A study by Cook et al demonstrated the potential of imaging brain targets using [^18^F]F-537-Tz in combination of antisense oligonucleotide conjugated TCO (ASO-TCO) in a pretargeted approach in rats. Rats were first injected with ASO-TCO into the intrathecal space followed by an intravenous injection [^18^F]F-537-Tz which resulted in higher uptake of tracer in brain with a brain to muscle ratio of 1.27 ± 0.09 compared to naïve rats (1.00 ± 0.02) or TCO-negative Malat1 ASO (1.01 ± 0.05) control group, demonstrating the brain penetration of [^18^F]F-537-Tz and a successful click reaction in brain. Despite these promising results, the radiotracer demonstrated slow kinetics in non-human primates, and PET imaging did not show specific binding in the brain (Cook et al. 2022).

In a recent study, Shalgunov et al reported a comparison between [^18^F]F-537-Tz and N-(3-[^18^F]fluoro-5-(1,2,4,5-tetrazin-3-yl)benzyl)propan-1-amine, for imaging of Aβ plaques in rats pretargeted with TCO-Peptobrush. While both radiotracers showed similar uptake N-(3-[^18^F fluoro-5-(1,2,4,5-tetrazin-3-yl)benzyl)propan-1-amine showed superior clearance from brain compared to [^18^F]F-537-Tz resulting in a higher image contrast (Shalgunov et al. 2024).

Other small molecules and nanoparticles have also been used in pretargeted imaging. One example is TCO-conjugated bisphosphonates in combination with ^99m^Tc-labeled tetrazine or ^177^Lu-labeled Tz for the imaging or therapy of bone diseases, respectively. When assessed in vivo the pretargeting approach showed selective and high uptake in the knee (20.07 ± 9% ID/g) and shoulder (16.2 ± 8% ID/g) (Yazdani et al. 2016). Recently, Zeglis et al. translated the approach to image osteodestructive lesions in dogs using [^64^Cu]Cu-SarAr-Tz, demonstrating the feasibility of in vivo pretargeting in larger animals with bigger blood volume (Maitz et al. 2022).

Pretargeting studies in mice injected with mesoporous silica nanoparticles (MSNs) conjugated with TCO and s-TCO, followed by Palacios et al. (1993) C-labeled tetrazine, resulted in a significantly higher uptake of activity in the lungs compared to the control group (Denk et al. 2016). Notably, MSNs conjugated to TCO outperformed those conjugated to s-TCO, even though both modified MSNs contained similar dienophile loading. This again highlights the effect of lower stability of s-TCO for in vivo applications. In another study, supramolecular nanoparticles (SNP) encapsulating TCO-grafted molecular building blocks, which disassemble once the SNPs are retained in the tumor via the enhanced permeability and retention (EPR) effect, were used in pair with ^64^Cu -labeled Tz. Pretargeted imaging studies showed accumulation and retention of radioactivity primarily in the tumors and livers of U87MG glioblastoma tumor-bearing mice. Although improved tumor uptake was observed in comparison to the traditional nanoparticle-based platform, high uptake of nanoparticles in the liver remains a disadvantage (Hou et al. 2016).

Besides imaging, pretargeting approach involving TCO-Tz ligation has also been applied in radioligand therapy. Pretargeted radioimmunotherapy (PRIT) is a promising strategy for delivering targeted cytotoxic radiation to solid tumors to induce therapeutic effects while minimizing toxicity to healthy tissues. In 2013, Rossin et al. used a PRIT strategy with a CC49-TCO conjugate to deliver a β-emitter [^177^Lu]Lu-DOTA-PEG_11_-Tz in LS174T-tumor-bearing mice. In this study, a galactose-albumin-based clearing agent was injected twice at 30h and 48h to enhance the clearance of excess mAb-TCO conjugate from the circulation before the injection of the radiotracer. Two hours later, [^177^Lu]Lu-DOTA-PEG11-Tz was injected. Using a clearing agent resulted in a twofold increase in tumor uptake and a 125-fold improvement in the tumor-to-blood ratio, which is crucial for reducing dose-limiting hematotoxicity in therapy (Rossin et al. 2013b). Dosimetry calculations suggested that the pretargeting system could allow for an eightfold higher tumor-absorbed dose in mice compared to the conventional approach (Poty et al. 2019). Encouraged by this, several PRIT studies with radionuclide-labeled tetrazine have been performed (Yazdani et al. 2016; Li et al. 2010b; Houghton et al. 2017; Membreno et al. 2018; Shah et al. 2017; Lappchen et al. 2017). However, only a few studies have reported the therapeutic efficacy of PRIT.

Houghton et al. demonstrated the use of a TCO-5B1 anti-CA19.9 mAb paired with ^177^Lu-labeled tetrazine for therapy in a BxPC3 mouse model of pancreatic cancer. TCO-5B1 was administered first followed 72h after by the injection of [^177^Lu]Lu-DOTA-PEG_7_-Tz at varying doses (400, 800, and 1,200 μCi). At higher doses of radioligand, a significant delay in tumor growth and regression of tumors was observed when compared to the control groups. (Houghton et al. 2017) Membreno et al. also reported a longitudinal PRIT study with the same Tz radioligand [ (Hou et al. 2016) Lu]Lu-DOTA-PEG_7_-Tz and a TCO-huA33 mAb in mice bearing SW1222 xenografts. The study showed a substantial decrease in tumor volume and a 100% survival rate for the treatment group, while all mice in the control group died during the course of the study (Membreno et al. 2018). Rondon et al. reported a PRIT study in a disseminated orthotopic model of peritoneal carcinoma (A431-CEA-Luc). Intraperitoneal injection of TCO-35A7 mAb followed 24h later by [^177^Lu]Lu-DOTA-PEG_7_-Tz demonstrated a significant delay in tumor growth in the PRIT cohort compared to the control. (Rondon et al. 2019). Poty et al. investigated the effectiveness of ^225^Ac-PRIT ([^225^Ac]Ac-DOTA-PEG_7_-Tz paired with 5B1-TCO) in both subcutaneous and orthotopic models of CA19.9-positive PDAC xenografts. The median survival of mice treated with either conventional radioimmunotherapy (RIT) or PRIT at the highest injected dose (37 kBq) was similar, indicating the effectiveness of PRIT. Nevertheless, a higher incidence of haematotoxicity was observed in mice treated with conventional RIT. In contrast, the maximal tolerated dose (MTD) was not reached in PRIT cohort allowing for higher injected activities in future investigations (Timperanza et al. 2023).

Keinänen et al. conducted a pretargeted theranostic study utilizing the copper-64 and copper-67 radioisotopes, where copper-64 was used for imaging and copper-67 for therapy. In a murine model of human colorectal carcinoma, therapeutic studies were conducted with varying doses of huA33-TCO and [^67^Cu]Cu-MeCOSar-Tz. Studies have revealed a dose-dependent therapeutic response, with a reduction in tumor volume and an increased median survival time for the highest dose of [^67^Cu]Cu-MeCOSar-Tz (55.5 MBq) in both the RIT and PRIT cohorts. However, mice injected with a fractionated dose of [^67^Cu]Cu-MeCOSar-Tz (2 × 27.8MBq) in a PRIT setting exhibited less hematological toxicity without compromising therapeutic efficacy. Furthermore, when [^64^Cu]Cu-MeCOSar-Tz was integrated into a theranostic approach, a correlation was observed between the tumor uptake of [^64^Cu]Cu-MeCOSar-Tz and the therapeutic response to [^67^Cu]Cu-MeCOSar-Tz. Mice with higher tumor uptake of [^64^Cu]Cu-MeCOSar-Tz showed tumor remission, whereas low tumor uptake led to tumor regrowth. This highlights the advantage of theranostic pair in predicting responders and non-responders (Keinanen et al. 2020).

#### ^18^F-labeled TCO in pretargeted imaging

**Fig. 10:**
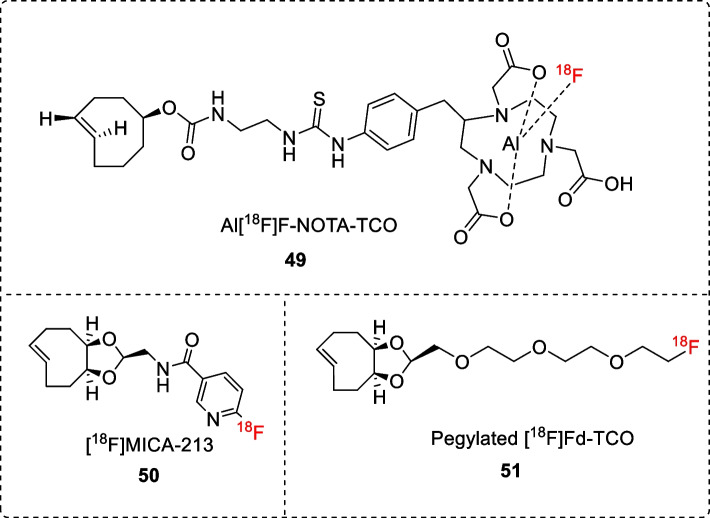
^18^F-labeled TCO and d-TCO derivatives used in pretargeted imaging

In contrast to radiolabeled Tz, radiolabeled TCO has been explored less for pretargeted imaging. Nevertheless, many studies have emerged in the past years. [^18^F]F-TCO was first evaluated for pretargeted PET imaging of the brain by Wyffels et al (2014). Although the study revealed the brain penetration of [^18^F]F-TCO, it was rapidly metabolized in both the plasma and brain with indications of further defluorination at later time points, thus rendering it unsuitable for in vivo use (Wyffels et al. 2014). Further efforts by Ruivo et al. to improve the stability of ^18^F-labeled TCO involved the incorporation of a 1,4,7-triazacyclononane-*N*,*N*’,*N*″-triacetic acid (NOTA) chelator as a linker and a radiolabeling method by complexation of Al[^18^F]F (Fig. 10; 49). The tracer showed improved in vivo stability, with 51.9 ± 5.16% of intact tracer remaining after 60 min. Moreover, *the cis*-isomer was identified as the only main radiometabolite and no defluorination was observed. In vivo pretargeted imaging of LS174T colon cancer xenografts in mice treated with Tz-conjugated CC49-antibody showed clear delineation of the tumor when compared to the control group. However, high nonspecific activity was observed in the abdominal region owing to the hydrophobicity of the TCO-core (Ruivo et al. 2019). Recently, radiolabeled d-TCOs have garnered attention for pretargeted imaging because of their fast kinetics, increased hydrophilicity, and improved stability. Billaud et al. synthesized a pegylated [^18^F]F-d-TCO (Fig. 10; 51), which was stable in PBS with 94% of the intact tracer remaining after 2 h at 37 °C. In addition, slow isomerization of [^18^F]F-d-TCO to the *cis-*isomer was observed in rat plasma, with 52% and 34% of the intact tracer remaining after 1 h and 2 h of incubation at 37 °C, respectively (Billaud et al. 2017), Despite favorable in vitro stability, the pharmacokinetics of the tracer were suboptimal, resulting in low absolute tumor uptake and prominent abdominal activity in the pretargeted approach (Billaud et al. 2017b). Subsequently, the ^18^F-labeled d-TCO amide derivative [^18^F]MICA-213 (Fig. 10; 50) was reported by Ruivo et al*. *In vitro, the tracer showed improved stability, with 79% of the intact tracer remaining after 2h of incubation in plasma at 37 °C. In a pretargeting study in mice bearing LS174T tumor xenografts, clear delineation of the tumor tissue was observed in the group pretreated with the CC49 Tz conjugate compared to the control group. Although increased tumor uptake was observed (1.36 ± 0.28% ID/g), a high background signal in the abdominal region resulted in a low tumor-to-background ratio (Ruivo et al. 2020). In all the above-mentioned examples, the high background due to abdominal uptake is a common factor that needs to be improved for further translation of radiolabeled TCOs in pretargeted imaging.

### Applications of TCO in targeted drug delivery

The click-to-release strategy holds great potential for enabling targeted drug delivery to cancerous tissues, offering an alternative approach to traditional antitumor drugs with their associated toxic effects and limited efficacy. By utilizing a locally triggered click-to-release mechanism, antitumor drugs can be specifically delivered or activated at the tumor sites, potentially reducing toxicity and enhancing therapeutic outcomes.

#### Tetrazine triggered drug cleavage from TCO conjugates

The Robillard group was the pioneer in demonstrating the efficacy of click-to-release in vivo. Following their initial study with r-TCO, the group demonstrated the use of a bifunctional click-cleavable TCO-linker (cTCO), which could be conjugated with an antibody and payload for targeted drug release in mice (Rossin et al. 2016). The cTCO-Dox prodrug was conjugated to the CC49 antibody, which targets the TAG72 antigen. ADC exhibited good in vivo stability with only a negligible amount of TCO deactivation (10% over 26h). In an LS174T tumor-bearing mouse model, the ^125^I-labeled ADC exhibited 30–40% ID/g tumor uptake at 30 h post-injection. Although the activation of ADC by Tz was selective, the release of free Dox was unsatisfactory, likely due to the rapid clearance of the trigger, DMT. However, modification of DMT with dextran to prolong its circulation half-life resulted in the release of up to 51.3% Dox 24 h after injection, emphasizing the importance of selecting a Tz with prolonged circulation (Rossin et al. 2016).

Subsequently, the Robillard group reported the use of an ADC approach utilizing diabody conjugates. Monomethyl auristatin E (MMAE) caged by a bifunctional cTCO was conjugated to the diabody through maleimide conjugation. ^125^I-labeled ADC showed fast clearance and selective uptake of 29% ID/g in LS174T tumors, which was comparable to the uptake value of CC49 mAb in the same tumor model. Additionally, the group replaced DMT-dextran with 3,6-bisalkyl-Tz-DOTA as the decaging activator. The introduction of a DOTA chelator into Tz resulted in a reduction in the clearance rate and an increase in the decaging yield. The reaction between cTCO-ADC and DOTA-Tz at the tumor site was examined in a blocking study. The study revealed no tumor uptake of ^177^[Lu]Lu-3,6-bispyridyl-Tz in the group in which 3,6-bisalkyl-DOTA-Tz was administered as a blocking agent before the radiotracer. In contrast, in the control group, where no Tz-activator was injected, high tumor uptake of ^177^[Lu]Lu-3,6-bispyridyl-Tz was observed. When examined for drug release levels in the tumor vs. other tissues, a 100-fold lower MMAE level was observed in the liver and plasma compared to the tumor in the group treated with cTCO-ADC followed by 3,6-bisalkyl-DOTA-Tz. Notably, therapeutic effect was demonstrated in two mouse LS174T and OVCAR-3 xenograft models treated with the cTCO-ADC conjugate and 3,6-bisalkyl-DOTA-Tz (Rossin et al. 2018). A multi-dose study with four cycles of ADC and Tz triggers resulted in tumor regression and survival of mice until the end of the study. This study highlights the therapeutic potential of small-molecule-triggered decaging reactions.

Mejia-Oneto et al. proposed a “catch and release” strategy to achieve a tissue-specific and controlled release of drugs in mice with soft tissue sarcoma. They used a TCO-carbamate-Dox prodrug with 75 times lower cytotoxic activity against HT1080 cells than that of Dox. Initially, the tetrazine-modified hydrogel was administered locally at the target site. This was followed by the intravenous injection of the prodrug, initiating a bioorthogonal reaction (the ‘catch’ step) as the prodrug circulated and reached the tumor, leading to its accumulation at the tumor site. Subsequent tautomerization and release of active Dox from TCO resulted in improved antitumor efficacy and reduced myelosuppression compared to the control group treated with free Dox (Mejia Oneto et al. 2016).

Czuban and coworkers applied a similar approach to concentrate and activate a systemically administered antibiotic prodrug by using a hydrogel platform containing multiple Tzs. This enabled the effective inhibition of both planktonic and biofilm growth of bacteria through activation of multiple dosages of the prodrug (Czuban et al. 2018).

Subsequently, Rozyen and coworkers introduced the click-activated protodrug against cancer (CAPAC) platform termed SQ3370 (Wu et al. 2021). A tetrazine-modified hyaluronate-based biopolymer (SQL70) was administered locally at the tumor site. This was followed by the intravenous administration of the TCO-modified prodrug with reduced cytotoxicity. In addition, the use of a bifunctional cTCO allowed for its modification with a cytotoxic payload on one end and a hydrophilic group on the other, thereby improving the pharmacokinetics of the prodrug. The lead candidate SQP33, consisting of a TCO functionalized with Dox and glycine, showed improved aqueous solubility and 83 times lower cytotoxicity than the parent compound. The maximum tolerated dose (MTD) of SQP33 with locally injected SQL70 in mice was found to be 19.1 times higher than that of free Dox. Additionally, pharmacokinetic studies in rats revealed that multiple doses of the SQP33 protodrug could be captured by a single injection of SQL70, while significantly reducing the systemic toxicity induced by free Dox. Moreover, the use of SQL70 and SQP33 in a syngeneic tumor mouse model with MC38 flank tumors resulted in significantly slower tumor growth and extended overall survival (Wu et al. 2021). This approach holds great promise, as it allows the concentration and local activation of cytotoxic drugs, allowing the administration of higher therapeutic doses while ensuring an improved safety profile. The targeted drug delivery platform SQ3370 has already advanced to a first-in-human Phase II clinical trial in patients with advanced solid tumors (U.S. National Library of Medicine 2023). It should be noted, however, that injectable hydrogel-based activation is primarily limited to the treatment of resectable tumors due to the requirement for hydrogel installation at the target site.

Applications of Tz-triggered release have been explored in various contexts to achieve spatiotemporally controlled prodrug activation. Chen et al. reported a phosphorylated self-assembly tri-peptide lysine-tyrosine-phenylalanine (KYF), conjugated with Tz as a targeting vector for cancer cells. Upregulation of phosphatase in cancer cells led to enzyme-instructed supramolecular self-assembly (EISA), resulting in the specific accumulation of Tz within cancer cells. In vivo treatment studies in mice bearing HeLa tumor xenografts with a TCO-Dox in combination with Tz-conjugated KYF demonstrated a selective release of Dox within the cancer cells, resulting in a reduced tumor volume when compared to group treated with saline (Yao et al. 2018).

In another example, fluorescently labeled magnetic iron oxide nanoparticles were used for image-guided prodrug activation. The nanoparticles were decorated with both Tz and a fluorophore. Fluorescence imaging confirmed the specific accumulation of nanoparticles in MDA-MB-231 breast cancer cells. Subsequent addition of TCO-Dox prodrug led to an efficient release of the active Dox (Khan et al. 2016).

In another study, the TCO-Dox prodrug and tetrazine were separately encapsulated within micelles that are sensitive to low pH and matrix metalloproteinase 2 (MMP-2), which are characteristic of the tumor microenvironment (TME). Upon systemic administration in mice with 4T1 tumors, the nanoparticles dissociated specifically in the tumor cells, leading to the selective activation of the prodrug in TME and inhibition of tumor development, while demonstrating lower toxicity in non-target tissues (Zuo et al. 2020).

Furthermore, the controlled in vitro and in vivo activation of TCO-caged cytotoxic T cells was demonstrated. T cells were caged using a cTCO protecting group at a key lysine residue essential for T cell activation, which was restored in the presence of Tz. The use of cTCO with a polar substituent enhanced the solubility of the modified peptide and increased its decaging yield. This approach was successfully implemented using two different epitopes, indicating its potential as a generic application for lysine-sensitive T-cell receptors (Gracht et al. 2018). In a similar approach, siRNA molecules were caged with cTCO conjugated to nanoparticles. After nanoparticle-mediated accumulation of caged siRNA in cells, the addition of tetrazine resulted in bond cleavage and release of activated siRNA. Gene silencing of GFP and CDK8 was demonstrated using TCO-siRNA and tetrazine-triggered cleavage in targeted cells (Khan et al. 2017). These examples highlight the versatility of the TCO-tetrazine reaction in controlling cellular processes and modulating biological functions.

Cell-selective proteome labeling through puromycin inactivation has also been achieved using a click-to-release reaction (Du et al. 2017). An mAb-tetrazine conjugate (cetuximab-Tz) was used to target cells overexpressing EGFR. Subsequently, TCO-modified puromycin nucleoside was added to release puromycin into the target cells following the IEDDA decaging reaction. This enabled selective incorporation of puromycin into protein synthesis for proteomic analysis. This strategy allowed for selective labeling, even in heterogeneous co-cultured cells, with labeling observed exclusively in cells expressing the antigen.

The click-to-release reaction has also been used as an alternative method for purifying RNA (Agustin et al. 2016). A cleavable TCO linker was coupled to the RNA strand in the final coupling step. Subsequently, the TCO-coupled RNA strand was treated with 3,6,bispyridyl-tetrazine-modified agarose, allowing the selective trapping of the product. The use of Tz with slow elimination kinetics allowed the filtration of impurities from the resin prior to the cleavage of TCO, thereby generating a pure product without contaminants.

## Discussion and future perspectives

TCOs have emerged as indispensable tools in the field of chemical biology and nuclear medicine. Owing to its rapid kinetics and high selectivity towards tetrazines, TCO-Tz ligation and click to release reaction have become cutting-edge approaches in various chemical transformations, labeling of biomolecules, and the release of bioactive compounds (Table 1). Particularly, in nuclear medicine there has been a growing focus on radiolabeling of TCO or using it as a bioorthogonal tag to achieve specific imaging of biological targets and drug delivery.

**Table 1:** Overview of different TCO derivatives used in biomedical settings

Structure	Applications	Advantages	Limitations
*Trans*-cyclooct-4-enol derivatives			
	In vitro: Cellular imaging through genetic incorporation of TCO-based ncAA (Oliveira et al. 2017; Plass et al. 2012; Uttamapinant et al. 2015; Sakin et al. 2017)	Fast labeling Enables orthogonal labeling with azide-alkyne click chemistry	Unsatisfactory genetic incorporation of TCO-based ncAA (Plass et al. 2012) Difficult wash out leading to high non-specific fluorescence background (Nikic et al. 2014; Lang et al. 2012)
	In vivo: Bioorthogonal tag for pretargeted imaging and therapy (Rossin et al. 2013a, 2010, 2014, 2013b; Lumen et al. 2022; Shah et al. 2017; Edem et al. 2019; Altai et al. 2016; Duijnhoven et al. 2015) *Clinical trial* TCO conjugated to hu5B1 for imaging of pancreatic cancer^203^	Widely used in vivo stability of TCO-mAb complex (Rossin et al. 2013a)	Slow isomerisation of TCO tag in physiological conditions (Rossin et al. 2010) Relatively slow reaction kinetics
^18^F-labeled *Trans*-cyclooct-4-enol-derivatives			
	In vivo*:* Radiotracer: Pretargeted imaging (Wyffels et al. 2014)		Poor in vivo stability of radiotracer (Wyffels et al. 2014)
	Prosthetic group: ^18^F-labeled RGD (Li et al. 2010a; Selvaraj et al. 2011; Liu et al. 2013b) ^18^F-labeled Exendin-4 peptide (Wu et al. 2013; Wang et al. 2012) ^18^F-labeled PARP1 inhibitors (Keliher et al. 2011; Reiner et al. 2011)	Rapid labeling (5 min) with efficient conjugation (RCY = 90%) at rt in aqueous media (Li et al. 2010a) High specific activities of labeled peptides	High lipophilicity of TCO-Tz ligation product leading to high background (Li et al. 2010a)
	In vivo*:* Radiotracer pretargeted imaging (Ruivo et al. 2019)	Improved in vivo stability (Ruivo et al. 2019)	Low tumor uptake with high background (Ruivo et al. 2019)
Conformationally strained *trans*-cyclooctene (s-TCO) and derivatives			
	In vitro*:* Cellular imaging of tetrazine encoded GFP (Blizzard et al. 2015)	Fastest kinetics reported (Taylor et al. 2011; Darko et al. 2014)	Poor in vitro and in vivo stability (Rossin et al. 2013a; Lang et al. 2012) Moderate stability to prolonged storage (Fang et al. 2019) Synthesis lacks stereoselectivity (Taylor et al. 2011)
^18^F-labeled conformationally strained *trans*-cyclooctene (s-TCO) derivative			
	In vivo*:* Radiolabeled prosthetic group: ^18^F-labeled RGD (Wang et al. 2016, 2020) ^18^F-labeled neurotensin (Wang et al. 2020; Feng et al. 2020) ^18^F-labeled exendin (Wang et al. 2020)	Enhanced kinetics enables the use of stable tetrazines for increased stability of labeled conjugate (Wang et al. 2020)	Low stability of radiotracer (Wang et al. 2016)
*cis*-dioxolane-fused *trans*-cyclooctenes (d-TCO) and derivatives			
	In vitro*:* Cellular imaging of tetrazine encoded GFP (Darko et al. 2014)	Fast kinetics and increased hydrophilicity (Darko et al. 2014) Short diastereoselective synthesis	
^18^F-labeled *cis*-dioxolane-fused *trans*-cyclooctenes (d-TCO) and derivatives			
	In vivo*:* Radiotracer Pretargeted imaging (Billaud et al. 2017b)		Fast in vivo metabolisation (Billaud et al. 2017) Low absolute tumor uptake (Billaud et al. 2017b)
	In vivo*:* Radiotracer Pretargeted imaging (Ruivo et al. 2020)	Improved plasma stability (Ruivo et al. 2020) Brain penetration (Ruivo et al. 2020)	Moderate lipophilicity and high background signal (Ruivo et al. 2020)
Axial-5-hydroxy-*trans*-cyclooctene (a-TCO)			
	In vitro*:* Cellular imaging (Pigga et al. 2021)	Short diastereoselective synthesis Improved hydrophilicity with rapid wash out from cells of a-TCO fluorophore conjugate Favorable balance reactivity vs stability	
Strained aziridine-fused *trans*-cyclooctene (Aza-TCO)			
	In vitro*:* Cellular imaging (Siegl et al. 2018)	Fast kinetics Formation of fluorogenic click product allows straightforward detection (Siegl et al. 2018)	Long synthesis (Siegl et al. 2018)
Oxazolone fused-TCO (Ox-TCO)			
	In vitro*:* Cellular imaging through genetic incorporation (Kozma et al. 2016)	Improved hydrophilicty (Kozma et al. 2016)	Unsatisfactory genetic incorporation Complex synthesis with formation of inseparable isomers (Hilderbrand et al. 2015)
4,6-dioxo-TCO (DO-TCO)			
	In vitro*:* Cellular imaging through genetic incorporation (Kozma et al. 2016)	Improved hydrophilicity Enhanced wash out TCO-conjugates with low background in fluorescence imaging (Kozma et al. 2016)	Slow kinetics (Hilderbrand et al. 2015) Complex synthesis (Hilderbrand et al. 2015)
*trans-*5-oxocene (Oxo-TCO)			
	In vitro*:* Cellular imaging of tetrazine encoded GFP (Lambert et al. 2017)	Improved hydrophilicity and kinetics (Lambert et al. 2017)	Inseparable isomers (Lambert et al. 2017) Complex synthesis (Lambert et al. 2017)
^18^F-labeled *trans-*5-oxocene (Oxo-TCO)derivative			
	In vivo*:* Radiolabeled prosthetic group: ^18^F-labeled neurotensin (Wang et al. 2019)	Lower non specific uptake of radiolabeled peptide (Wang et al. 2019)	Use of inseparable isomers (Wang et al. 2019)
*Trans*-cyclooct-2-en-1-yl carbamate (r-TCO)			
	In vitro*:* RNA purification (Agustin et al. 2016) Image guided TCO-caged prodrug activation (Khan et al. 2016) Targeted proteome labeling (Du et al. 2017) In vivo: Targeted release of TCO-caged cytotoxic drugs, enzymes, antibiotics and proteins (Mejia Oneto et al. 2016; Rossin et al. 2018; Czuban et al. 2018; Yao et al. 2018; Zuo et al. 2020)	High stability (Nikic et al. 2014)	Slow kinetics High lipophilicity of TCO led to low solubility of conjugated drugs (Wu et al. 2021)
Click-cleavable TCO (cTCO)			
	In vivo: Targeted release of TCO caged cytotoxic drugs (Rossin et al. 2016; Wu et al. 2021) Activation of TCO-caged cytotoxic T cells (Gracht et al. 2018) Activation of siRNA nanodrug (Khan et al. 2017) Clinical trial: Targeted delivery of protodrug cTCO-Doxorubicin in solid tumors^193^	Additional site for functionalization (Rossin et al. 2016)	Long synthesis (Rossin et al. 2016)
Dioxolane-fused cleavable TCO (dcTCO)			
	In vitro: Targeted release of dcTCO prodrug (Kuba et al. 2023)	Short diastereoselective synthesis (Kuba et al. 2023) Increased hydrophilicity and reactivity (Kuba et al. 2023) High stability	

Underlined is described the application of each compound. For example, as a radiotracer or as a prosthetic group

Although recent developments are promising, several key challenges must be addressed in the future. While many TCOs have been developed and efforts have been made to establish more feasible synthetic pathways, functionalized TCOs remain relatively inaccessible compared with their tetrazine counterparts. For instance, the more hydrophilic oxo-TCO was used as a diastereomeric mixture. Although flow-photoisomerization has facilitated the synthesis of different TCOs, the scalability and ease of purification of the produced diastereomers remains challenging.

Stability poses another challenge for the use of TCOs. A slow isomerization of TCO in vivo is acceptable considering its fast kinetics. Moreover, non-crystalline derivatives of strained TCOs tend to isomerize during prolonged storage. However, storing these derivatives as stable silver(I) (Ag(I)) metal complexes can extend their shelf life, which can be readily dissociated upon the addition of NaCl (Fang et al. 2019). Storing TCOs as Ag(I)-TCO complexes might offer a solution for long-term storage.

Moreover, the stability requirements of the bioorthogonal reaction partners may vary depending on their specific roles. Bioorthogonal tags require higher stability as they reside longer in biological conditions throughout the duration of an experiment, whereas reporter probes only have to be stable during the labeling phase. Consequently, *trans*-cyclooct-4-enol has often been the bioorthogonal tag of choice for in vivo applications, as it is less prone to isomerization than s-TCO and d-TCO. For live-cell applications, faster-reacting s-TCO and d-TCO are highly valuable as reporting probes, as only a short incubation time is required owing to their rapid kinetics. Several studies have used these probes for cellular and in vivo experiments. However, the higher lipophilicity of the TCO and s-TCO probes also led to significant nonspecific labeling, necessitating intensive washing steps. Novel *trans*-5-oxocene and a-TCOs have shown improvement owing to their increased hydrophilicity, but only a few studies have been reported to date using these compounds. Overall, there is a need to develop TCO derivatives with enhanced stability and increased hydrophilicity while still displaying rapid kinetics under cellular or in vivo conditions.

Several radiolabeled TCOs have been reported in the past decade. TCO’s are stable under nucleophilic substitution reaction conditions and have been directly labeled with ^18^F. Radiolabeled TCOs function as prosthetic groups in the field of radiochemistry and facilitate rapid and selective labeling of proteins and peptides under physiological and mild conditions, enabling the imaging of diverse molecular targets. The use of radiolabeled TCOs for pretargeted imaging and therapy has been rather limited when compared to radiolabeled tetrazines. Several radiolabeled tetrazines have been developed for pretargeted imaging and therapy, achieving good tumor to non-tumor ratios. While tetrazines were often labeled using chelation chemistry in the past, new direct ^18^F-labeling strategies have also emerged in the past years expanding the bioorthogonal toolbox (Garcia-Vazquez et al. 2021, 2022). The use of TCO-Tz ligation has revolutionized the field of pretargeted imaging, resulting in specific tumor uptake in tumor tissue and higher image contrast, ultimately leading to reduced radiation burden and toxicity. Several preclinical studies have employed this approach for tumor imaging. Recently, with the development of direct radiofluorination methods, ^18^F-labeled brain permeable tetrazines for imaging studies have emerged (Syvanen et al. 2020; Shalgunov et al. 2023, 2024). Nonetheless, clinical translation of the pretargeting approach based on IEDDA is still lagging behind. A noteworthy milestone is the recently FDA approved human clinical trial for PET imaging of pancreatic cancer patients, using ^64^Cu -Tz-SarAr and hu5B1-TCO through a pretargeting approach (U.S National Library of Medicine 2023).

Nevertheless, the reverse approach, in which a TCO is radiolabeled and tetrazine is conjugated to a biological vector, enables the use of fast-reacting TCOs when rapid reaction kinetics are paramount. Additionally, inherent lipophilicity of the TCOs could also facilitate the permeation of the blood–brain-barrier for pretargeted imaging of targets in the brain. For instance, ^18^F-labeled d-TCO (50) showed brain uptake followed by a fast washout of activity (Ruivo et al. 2020). Although examples of pretargeted imaging studies using ^18^F-labeled TCO and d-TCO radiotracers are emerging, substantial improvements in tracer kinetics and biodistribution are required. The inherent hydrophobic core of these probes often leads to high abdominal uptake, compromising the image contrast. Therefore, the challenge lies in developing stable and hydrophilic TCOs with high reactivity and functional handles that allow modulation of pharmacokinetics to reduce nonspecific accumulation.

Additionally, pretargeted radioimmunotherapy through TCO-Tz ligation is an emerging field with significant therapeutic potential. Treatment of solid tumors require higher radiation doses which often results in higher off-target toxicity in radiation sensitive tissues, such as the bone marrow. Pretargeting through TCO-Tz ligation enables the administration of higher radiation doses given the fast clearance of radiolabeled compound which results in lower toxicities. PRIT studies have shown that the dosimetry received by bone marrow is halved in comparison to conventional approach (Membreno et al. 2018). This is particularly important in the case of targeted alpha-particle therapy, which aims to deliver radioisotopes emitting alpha-particles (highly cytotoxic) to tumors (e.g., Astatine-211). Although, the PRIT studies described to date have used radiolabeled tetrazines, with the growing interest in the field, and the increasing availability of radiolabeled TCOs with distinct pharmacokinetic profiles, we believe that TCO-derivatives labeled with theranostic radioisotopes will emerge in the near future.

Similarly, decaging reactions using TCO-cleavable linkers have shown great potential for targeted drug delivery. This approach expands the range of targets for ADC therapy beyond those that rely on the existing intracellular activation mechanisms. Studies aimed at expanding the number of functional groups that can be decaged from TCO have been conducted. This enables the decaging of a variety of functional groups, expanding the scope for the unmasking of drugs, amino acid residues, proteins, and other biomolecules. These applications demonstrate the broad usefulness of the TCO-tetrazine reaction beyond bioorthogonal chemistry, paving the way for innovative strategies in cell labeling, RNA synthesis, and controlled release of bioactive compounds. A human clinical trial with a hydrogel-based platform SQ3370 for the release of a cytotoxic drug has already moved towards phase II. Furthermore, studies aimed at enhancing the therapeutic efficacy of ADCs for targeted tumor treatment have been reported.

## Conclusions:

In our review, we explored different types of TCOs and discussed recent advancements in the synthesis of their derivatives, as well as their reactivity and stability profiles. We showcase the versatility of TCOs through examples of their applications in ligation and release chemistry, where they have proven to be powerful tools for targeted imaging and drug delivery platforms. The use of TCO as a reactant in IEDDA reactions holds immense potential for the study of various biological targets and has substantial implications in nuclear medicine for the development of improved imaging and therapy platforms. Moreover, the potential of TCO-based ligation reactions for nuclear imaging and therapy is still unfolding and potentially revolutionize the theranostic approach.

## Data Availability

Not applicable.

## Abbreviations:

TCO: *trans-*Cyclooctene
HOMO: Highest occupied molecular orbital
CuAAC: Copper-catalyzed azide-alkyne cycloaddition
SPAAC: Strain-promoted azide-alkyne cycloaddition
IEDDA: Inverse electron-demand Diels–Alder
LUMO: Lowest unoccupied molecular orbital
EWG: Electron-withdrawing group
FMO: Frontier molecular orbital
EDG: Electron-donating group
CCO: *cis*-Cyclooctene
s-TCO: Strained cyclopropane fused TCO
d-TCO: Cis dioxolane fused TCO
DFT: Density functional theory
BCN: Bicyclononynne
Tag silica: Tosic-acid-functionalized silica gel
FEP: Fluorinated ethylene propylene
mAb: Monoclonal antibody
k_2_: Second order rate constant
GFP: Green fluorescent protein
Aza-TCO: Strained aziridine-fused *trans*-cyclooctene
ncaa: Noncanonical amino acid
Ox-TCO: Oxazolon-fused *trans*-cyclooctene
DO-TCO: 4,6-Dioxo *trans*-cyclooctene
SPECT: Single-photon emission computed tomography
PET: Positron emission tomography
RCY: Radiochemical yield
PARP1: Poly(ADP-ribose)polymerase 1
HNSCC: Head and neck squamous cell carcinoma
TfR: Transferrin receptor
BBB: Blood-brain barrier
Aβ: β-Amyloid
MSNs: Mesoporous silica nanoparticles
SNP: Supramolecular nanoparticle
EPR: Enhanced permeability and retention
PRIT: Pretargeted radioimmunotherapy
RIT: Radioimmunotherapy
MTD: Maximal tolerated dose
NOTA: 1,4,7-Triazacyclononane-*N*,*N*’,*N*″-triacetic acid
Dox: Doxorubicin
DMT: Dimethyltetrazine
r-TCO: *trans*-Cyclooct-2-en-1-yl carbamate
ADC: Antibody drug conjugate
cTCO: Click-cleavable TCO
C_2_TCO: Cleavable C_2_-symmetric *trans*-cyclooctene
dcTCO: Dioxolane-fused cleavable TCO
TIPS: Triisopropylsilyl
CA4: Combretastatin A4
MMAE: Monomethyl auristatin E
EISA: Enzyme-instructed supramolecular self-assembly
CAPAC: Click-activated protodrug against cancer
MMP-2: Matrix metalloproteinase 2
TME: Tumor microenvironment
